# Quantitative measurements of kinetochore protein copy numbers via FCS-calibrated molecular counting

**DOI:** 10.1083/jcb.202603185

**Published:** 2026-11-11

**Authors:** Yasuhiro Hirano, Yusuke Takenoshita, Akira Kitamura, Toru Hirota, Tatsuo Fukagawa

**Affiliations:** 1 Graduate School of Frontier Biosciences, The University of Osaka, Suita, Japan; 2 The Cancer Institute, Japanese Foundation for Cancer Research, Tokyo, Japan; 3 Department of Chemistry, Faculty of Science, Ochanomizu University, Tokyo, Japan; 4 Faculty of Advanced Life Science, Hokkaido University, Sapporo, Japan; 5 PRIME, Japan Agency for Medical Research and Development, Tokyo, Japan

## Abstract

The kinetochore is a protein complex for faithful chromosome segregation. While its architecture has been characterized *in vitro* based on structural studies, the molecular organization of the kinetochore in living cells remains unclear. To understand the molecular organization of kinetochore proteins in cells, we applied fluorescence correlation spectroscopy (FCS), which enables absolute molecular counting based on its theoretical framework. This approach confirmed that CENP-C is present at roughly double the copy number compared with other components of the constitutive centromere-associated network such as CENP-T. We also analyzed the KMN network, specifically the Mis12 complex (Mis12C) and the Ndc80 complex (Ndc80C), and asked whether the recruitment of these outer kinetochore components relies on CENP-T and/or CENP-C. When either CENP-T or CENP-C lacks the Mis12C-binding domain, cells utilize the remaining domain as a redundant pathway to sustain Ndc80C recruitment. This study provides molecular abundance at the kinetochore with theory-grounded accuracy and offers insights into its structural organization in cells.

## Teaser

Hirano et al. study the molecular organization of functional kinetochores in living cells via FCS-calibrated molecular counting. Through this method, they quantify the kinetochore protein copy number per kinetochore and conclude that CENP-C and CENP-T redundantly maintain the amount of the Ndc80 complex at mitotic kinetochores.

## Introduction

The kinetochore—a large protein complex assembled on the centromere of each chromosome—is essential for faithful chromosome segregation (Fukagawa and Earnshaw, 2014; Westhorpe and Straight, 2013). In many organisms, the centromere is specified by the histone H3 variant CENP-A (Black and Cleveland, 2011; Palmer et al., 1987) and various proteins are assembled on the centromeric chromatin to form the kinetochore, which serves as a physical and functional bridge between chromosomes and spindle microtubules (Ariyoshi and Fukagawa, 2023; Fukagawa and Earnshaw, 2014; Hara and Fukagawa, 2018; McKinley and Cheeseman, 2016; Mellone and Fachinetti, 2021). Thus, understanding how the kinetochore is organized on the centromere is an important biological issue.

The kinetochore consists of two major subcomplexes: the constitutive centromere-associated network (CCAN) and the KMN network (KMN). In vertebrates, the CCAN comprises 16 proteins (CENP-C, -H, -I, -K, -L, -M, -N, -O, -P, -Q, -R, -S, -T, -U, -W, and -X) and constitutively localizes to the centromere throughout the cell cycle (Amano et al., 2009; Foltz et al., 2006; Hori et al., 2008; Izuta et al., 2006; Nishino et al., 2012; Okada et al., 2006). The KMN, composed of the Knl1 complex, Mis12 complex (Mis12C), and Ndc80 complex (Ndc80C), is recruited to the CCAN from late G2 phase to the onset of mitosis, forming the functional kinetochore required for spindle microtubule attachment (Alushin et al., 2010; Cheeseman et al., 2006; DeLuca et al., 2006; Hara and Fukagawa, 2017; McKinley and Cheeseman, 2016; Nagpal and Fukagawa, 2016; Pesenti et al., 2016). To understand the molecular architecture of the kinetochore, the structures of *in vitro* reconstituted CCAN and KMN complexes have been resolved by single-particle analysis with cryo-electron microscopy (Pesenti et al., 2022; Petrovic et al., 2016; Polley et al., 2024; Sakai et al., 2026; Takenoshita et al., 2024; Yatskevich et al., 2022; Yatskevich et al., 2024). While the high-resolution structural information of kinetochore subcomplexes is useful, such structural information is limited to understanding the kinetochore organization in cells because CCAN and KMN units may not uniformly exist in cells. In fact, previous studies suggest that the copy numbers of individual kinetochore components for CCAN and KMN are not equivalent (Johnston et al., 2010; Suzuki et al., 2015). Moreover, recent studies have shown that oligomerization of CENP-C and CENP-T is essential for functional kinetochore formation *in vivo* (Hara et al., 2023; Sissoko et al., 2024) and that Ndc80C is clustered on microtubules (Polley et al., 2023). These studies indicate that clarification of the molecular architecture of the kinetochore in cells is essential. Toward such a goal, determining how many proteins are assembled at the kinetochore in cells is a fundamental step. Although previous studies have tackled this issue using immuno-blotting combined with fluorescence microscopy (Johnston et al., 2010; Suzuki et al., 2015), the accuracy of this approach is limited because it relies on external standards to convert fluorescence intensity into protein copy number. Alternatively, it requires assumptions relating protein copy number to the measured intensity. Thus, a quantitative analysis of kinetochore protein copy numbers using a theory-grounded method is needed.

Fluorescence correlation spectroscopy (FCS) has the potential to meet the requirement. FCS determines the diffusion coefficient, the brightness of fluorescent molecules, and the number of them in the observation volume by measuring fluorescence-intensity fluctuations and fitting the autocorrelation function (ACF) (Kitamura and Kinjo, 2018). However, its straightforward application to quantifying the number of kinetochore proteins is limited because they exhibit extremely low mobility (Hori et al., 2003; Nagpal et al., 2023; Watanabe et al., 2022). This restricted mobility of kinetochore proteins makes photobleaching more likely during data acquisition, compromising the accuracy of ACF fitting and ultimately hindering reliable copy number determination. To overcome these limitations of conventional FCS for immobile or low-mobility proteins, a method known as FCS-calibrated molecular number quantification has been proposed (Brunner et al., 2025; Otsuka et al., 2023; Politi et al., 2018). Because this approach is grounded in theoretical principles and makes no assumptions, it enables accurate quantification of protein abundance in cells.

In this study, we applied FCS-calibrated molecular counting to kinetochore proteins, combined with CRISPR-based gene editing in chicken DT40 cells. Using this strategy, we succeeded in measuring kinetochore protein copy numbers. These analyses contribute to our understanding of kinetochore organization in cells.

## Results and discussion

### The copy number of CCAN components within DT40 cells

**Figure 1.**
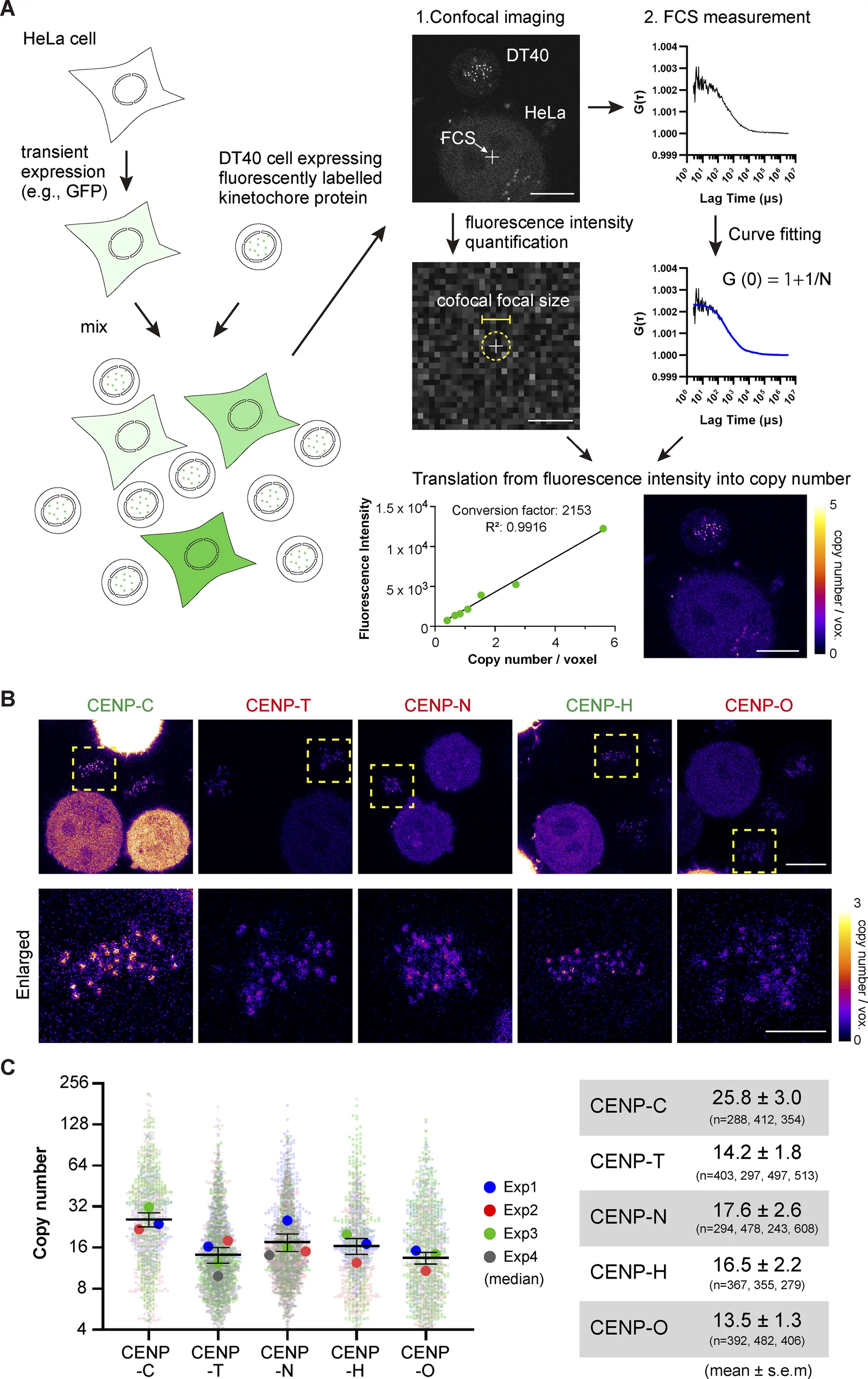
**Copy numbers of CCAN components. (A)** Schematic representation of FCS-calibrated protein copy number quantification procedure used in this study. A fluorescent protein was transiently expressed in HeLa cells, and the cells were mixed with DT40 cells expressing a fluorescently labelled kinetochore protein. After acquiring a confocal image and measuring FCS, the average fluorescence intensity (AFI) at the FCS measurement site, indicated by a white cross, and protein copy number were determined. The conversion factor (CF) to translate the fluorescence intensity into protein copy number was obtained by linear regression. Finally, the fluorescence intensity of the confocal image was translated into protein copy number. Scale bars in the top, middle, and bottom images represent 10, 1, and 10 μm, respectively. **(B)** Representative copy number image of CCAN proteins. FCS-calibrated copy number images of the CCAN components indicated at the top of the panel are shown. The character colors at the top correspond to the colors of the tagged fluorescent proteins. An enlarged image of the yellow dashed-square region is shown at the bottom. A color scale indicating protein copy number per voxel is shown on the right. Scale bars: 10 and 5 μm for top and bottom, respectively. **(C)** Copy numbers of CCAN components per kinetochore. The copy number of CCAN components per kinetochore was quantified from three or four independent experiments and is plotted using distinct colors. Dark closed circles indicate the median value for each experiment. Horizontal lines represent the average of these medians ± standard error (SEM). The determined copy numbers and the number of kinetochores analyzed are presented on the right.

**Figure S1.**
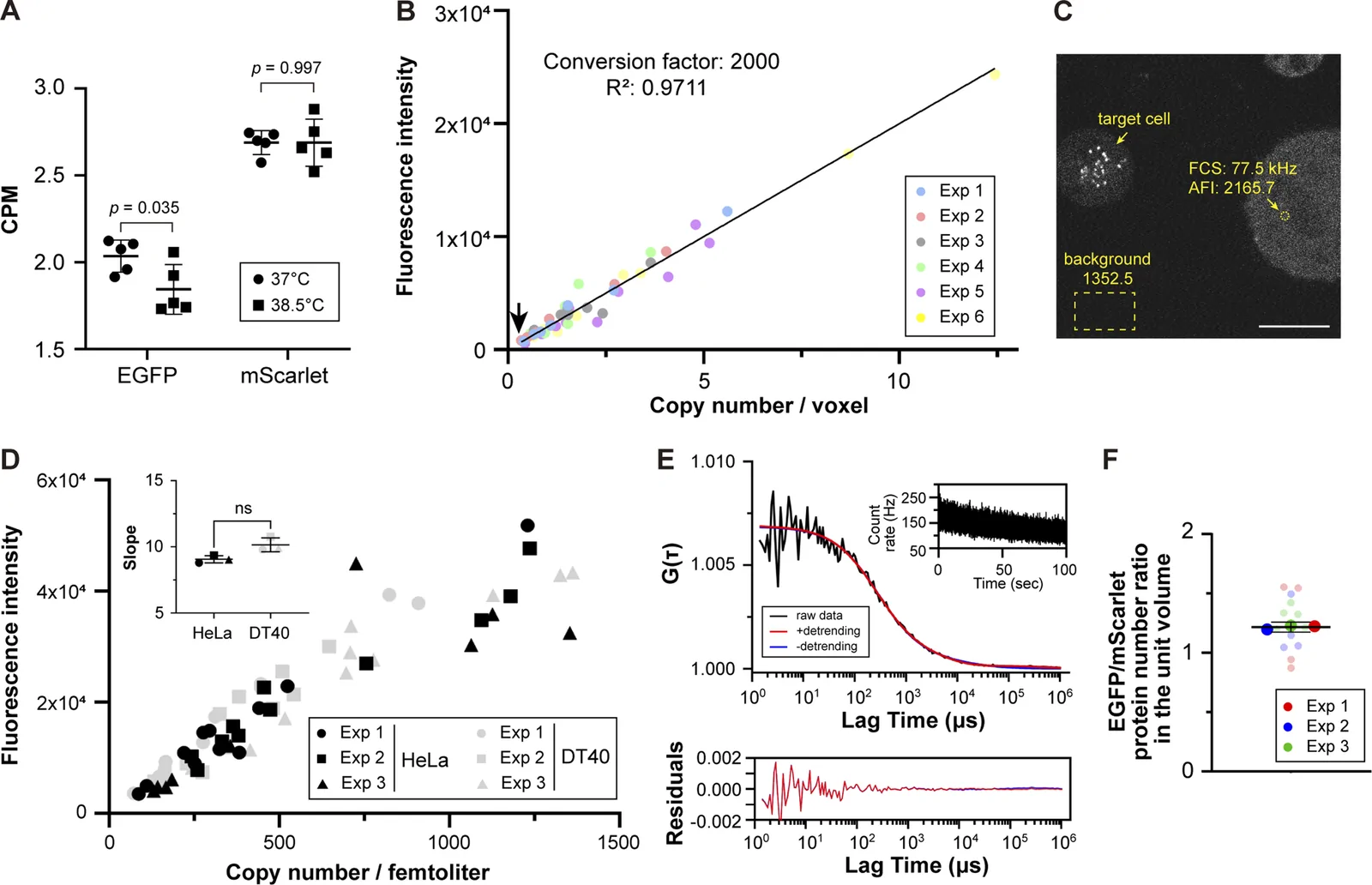
**FCS-calibrated kinetochore protein copy number quantification. (A)** Molecular brightness of fluorescence proteins at different temperatures. EGFP or mScarlet was transiently expressed in HeLa cells. Following transfection, HeLa cells were cultured overnight at either 37°C or 38.5°C to allow fluorescent protein maturation under different temperature conditions. Molecular brightness, equal to counts per molecule (CPM), was determined by FCS measurement, followed by fitting the autocorrelation curve. Horizontal lines represent mean ± SD from five cells in one experiment. Statistical significance was assessed using a two-tailed Welch’s *t* test. Data distribution was assumed to be normal, but this was not formally tested. The p value is shown at the top. **(B)** Translation of fluorescence intensity to protein copy number. The copy number of EGFP in the HeLa cells was determined by FCS, and the corresponding average fluorescence intensity (AFI) at the FCS measurement site was quantified. The copy number obtained by FCS was converted to the copy number per voxel in the confocal image, and the resulting values were plotted. Data from 50 cells across six independent experiments were plotted, and the relationship between AFI and protein copy number was assessed using linear regression. The slope (CF) and R^2^ value of the fitted line are shown. An arrow indicates the data shown in C. **(C)** S/N in FCS measurements and confocal imaging. The image corresponding to the data with the lowest copy number in the FCS analysis (the point indicated by the arrow in B) is shown. Scale bar: 10 μm. **(D)** A comparison of the standard curves obtained from HeLa and DT40 cells. The relationship between copy number and average fluorescence intensity of EGFP at the FCS-measured point was determined from three independent experiments in HeLa and DT40 cells. The slope values obtained from each experiment are plotted as an inset in the upper left. Horizontal lines in the inset represent the mean ± SD from three experiments. Data distribution was assumed to be normal, but this was not formally tested. Statistical significance was assessed using a two-tailed Welch’s *t* test. *ns* indicates no significant difference. **(E)** Detrending to normalize photobleaching during FCS measurement. FCS measurement of mScarlet protein was performed in the HeLa cell (upper-right inset). ACF obtained from the measurement (black) was fitted using the model either with (red) or without (blue) the stretched exponential term for detrending. Residuals after curve fitting are shown at the bottom. **(F)** The ratio of the copy numbers of EGFP and mScarlet per unit volume. To validate the copy numbers of EGFP and mScarlet within the identical observation volume, mScarlet-EGFP was expressed in HeLa cells, and its copy number was quantified by FCS in both the green and red channels. The copy number ratios obtained from five cells per experiment are plotted. Horizontal lines indicate the mean ± 95% CI across three independent experiments.

**Figure S2.**
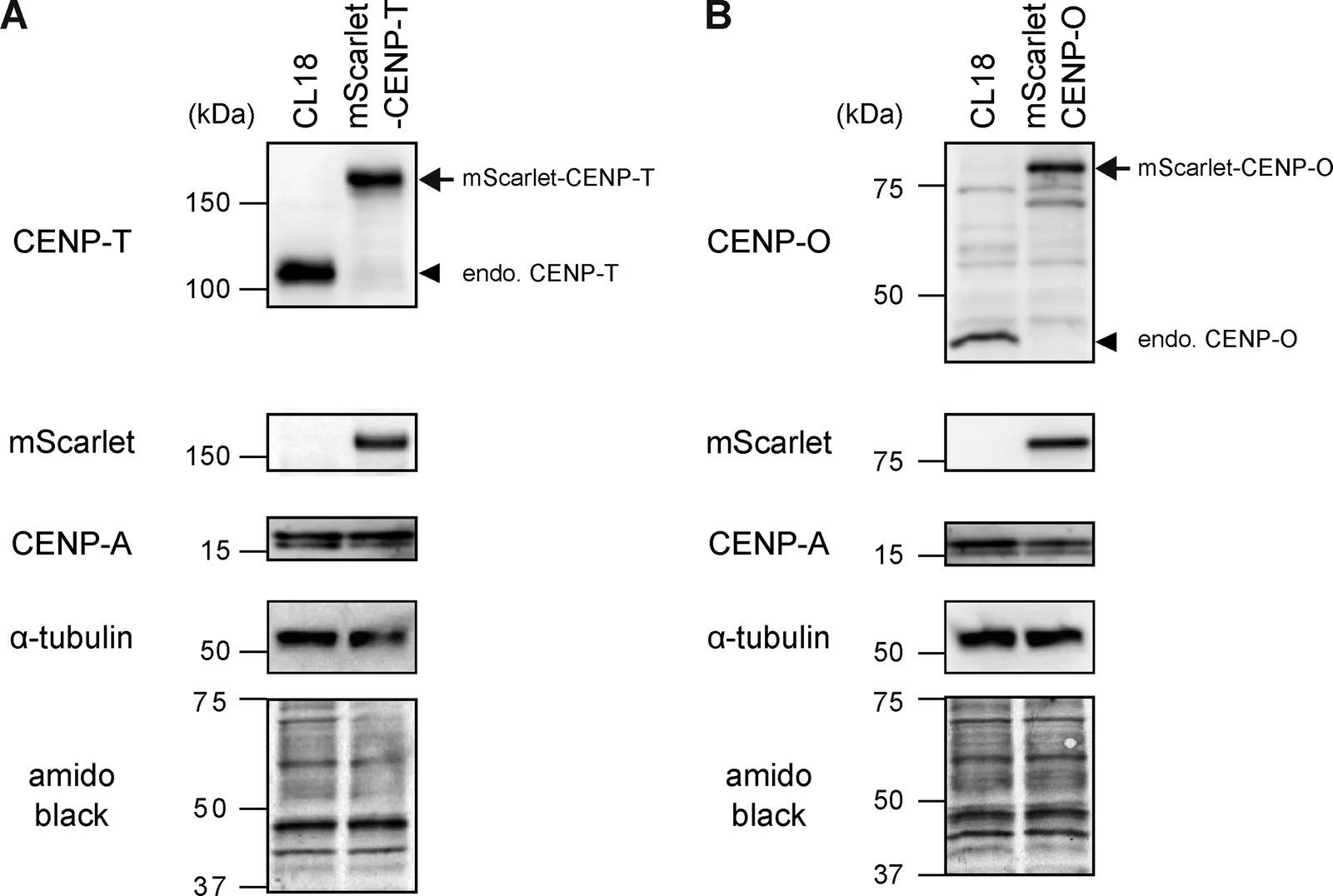
**Validation of protein expression levels of CCAN proteins used in this study. (A and B)** Endogenous CENP-T (A) or CENP-O (B) were replaced with fluorescently labeled counterparts. Protein levels of CCAN, CENP-A, and α-tubulin extracted from the parental cells (CL18) and cells expressing fluorescently labeled CCAN components were detected using antibodies indicated on the left. Protein molecular weight markers are indicated on the left. An amido black staining image for the loading control is shown at the bottom. The arrow and arrowhead represent fluorescently labeled and endogenous CCAN components, respectively. Source data are available for this figure: SourceData FS2.

We first examined the copy number of CCAN components using FCS-calibrated molecular number quantification (Fig. 1 A). The validity of this method is described in the Materials and methods and Fig. S1. For the quantification, we generated DT40 cell lines in which each kinetochore protein was replaced with an EGFP- or mScarlet-fused protein: cell lines expressing fluorescently labeled CENP-C, CENP-T, CENP-N, CENP-H, or CENP-O at expression levels comparable with their endogenous counterparts were established (Fig. S2; Fukagawa et al., 2001; Hara et al., 2023; Watanabe et al., 2019).

We determined the copy number of CCAN components at mitotic kinetochores because it would be technically difficult to distinguish the single kinetochore in interphase: (1) kinetochores across the chromosomes are clustered in interphase, and (2) the kinetochores built on the duplicated chromosome in S phase are not distinguishable due to the spatial resolution limit of confocal microscopy. The copy numbers of CCAN components at mitotic kinetochores were determined to be 25.8 ± 3.0, 14.2 ± 1.8, 17.6 ± 2.6, 16.5 ± 2.2, and 13.5 ± 1.3 for CENP-C, CENP-T, CENP-N, CENP-H, and CENP-O, respectively (Fig. 1, B and C). These values indicate a nearly equal stoichiometry among CCAN components, with the notable exception of CENP-C, whose copy number was roughly double that of the other CCAN components.

This stoichiometry of the CCAN components was comparable, whereas the copy numbers of CCAN components measured in this study are slightly lower than those in our previous study (Johnston et al., 2010). This difference likely arises from two factors: (1) differences in expression levels between constructs driven by the CMV promoter (previous study, except for the case of CENP-H-EGFP) and those under the control of native promoters (this study) and (2) variation in molecular brightness between GFP variants (S65T and EGFP) under different culture conditions, both of which compromise the accuracy of molecular number quantification. Because FCS provides an advantage for determining absolute molecular numbers and we carefully controlled experimental conditions, the values obtained in this study likely represent the native copy numbers of CCAN proteins.

Even under these measurement conditions, the copy number of CCAN components exhibited substantial variation across individual kinetochores. This variability likely arises from two factors: (1) differences in centromere size among chromosomes (Corda and Giunta, 2025) and (2) technical limitations in measurement. Kinetochore components assemble on the centromeric region marked by CENP-A, whose number varies among chromosomes in each cell. Regarding the second factor, some kinetochores remained in close proximity even in mitosis, which made them difficult to separate microscopically and segment accurately during the image analysis step. Nevertheless, this effect should be negligible because a stringent threshold was applied to minimize overlap, and a large number of kinetochores was quantified in each experiment. Thus, this variation likely reflects the different protein copy numbers per kinetochore.

Our measurement revealed that the copy number of CENP-C is approximately twice that of the other CCAN components, which is consistent with previous studies (Johnston et al., 2010; Suzuki et al., 2015). This suggests that CENP-C plays a distinct role in kinetochore assembly among CCAN components. The potential role of CENP-C in the kinetochore architecture will be discussed later.

### CENP-T and -C have redundant roles in loading the Mis12C

**Figure S3.**
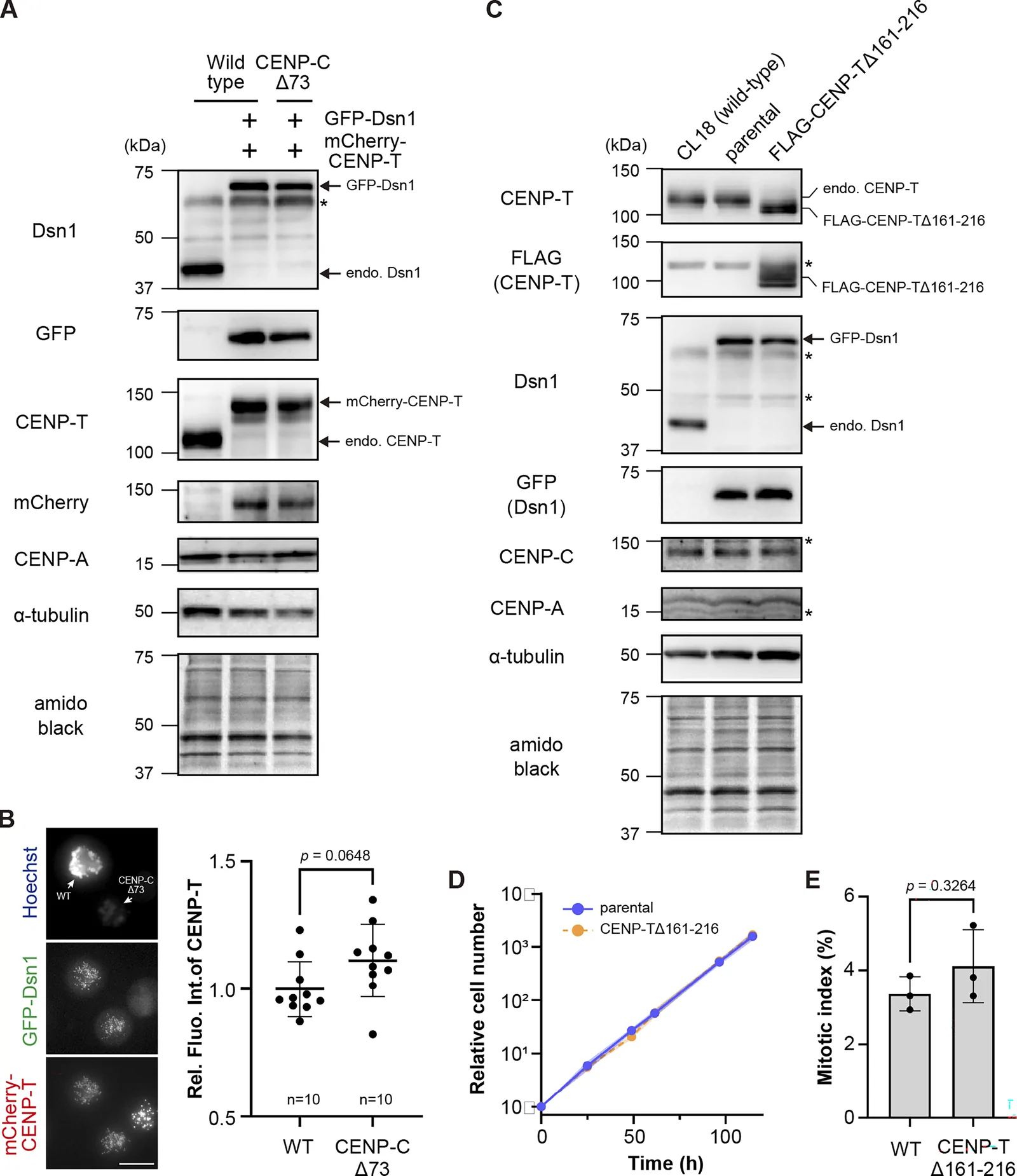
**Validation of cells used for the quantification of the copy number of Mis12C. (A)** Protein levels of EGFP-Dsn1 and mCherry-CENP-T. Endogenous CENP-T in CL18/EGFP-Dsn1 and CENP-CΔ73/EGFP-Dsn1 cells was replaced with mCherry-CENP-T. Protein levels of Dsn1, CENP-T, CENP-A, and α-tubulin were detected using antibodies indicated on the left. Protein molecular weight markers are indicated on the left. An amido black staining image for the loading control is shown at the bottom. An asterisk on the right indicates a nonspecific band. **(B)** CENP-T level at kinetochores remains unchanged in WT and CENP-CΔ73 cells. WT and CENP-CΔ73 cells were stained with Hoechst 33342 for long and short periods, respectively, to distinguish the two populations, and were then mixed. The average fluorescence intensity of mCherry-CENP-T at kinetochores was quantified in WT and CENP-CΔ73 cells and plotted. Statistical significance was assessed using a two-tailed Welch’s *t* test. The p value is shown at the top. Scale bar: 5 μm. **(C)** Protein levels of FLAG-CENP-TΔ161–216. Endogenous CENP-T in CL18/EGFP-Dsn1 was replaced with the FLAG-CENP-TΔ161–216 mutant. Protein levels of CENP-T, Dsn1, CENP-C, CENP-A, and α-tubulin were detected using antibodies indicated on the left. Protein molecular weight markers are indicated on the left. An amido black staining image for the loading control is shown at the bottom. Asterisks on the right indicate nonspecific bands. **(D)** Cell growth of CENP-TΔ161–216 mutant. Parental and CENP-TΔ161–216 mutant cells were cultured for 5 days, and cell numbers were measured at certain time points. Relative cell number, normalized to the starting point, was calculated from three independent experiments, and the mean ± SD is plotted. **(E)** Mitotic index of CENP-TΔ161–216 mutant. The percentage of mitotic cells in WT and CENP-TΔ161–216 cells was quantified across three independent experiments, and the mean ± SD is plotted. Statistical significance was assessed using a two-tailed Welch’s *t* test. Data distribution was assumed to be normal, but this was not formally tested. The p value is shown at the top. Source data are available for this figure: SourceData FS3.

**Figure 2.**
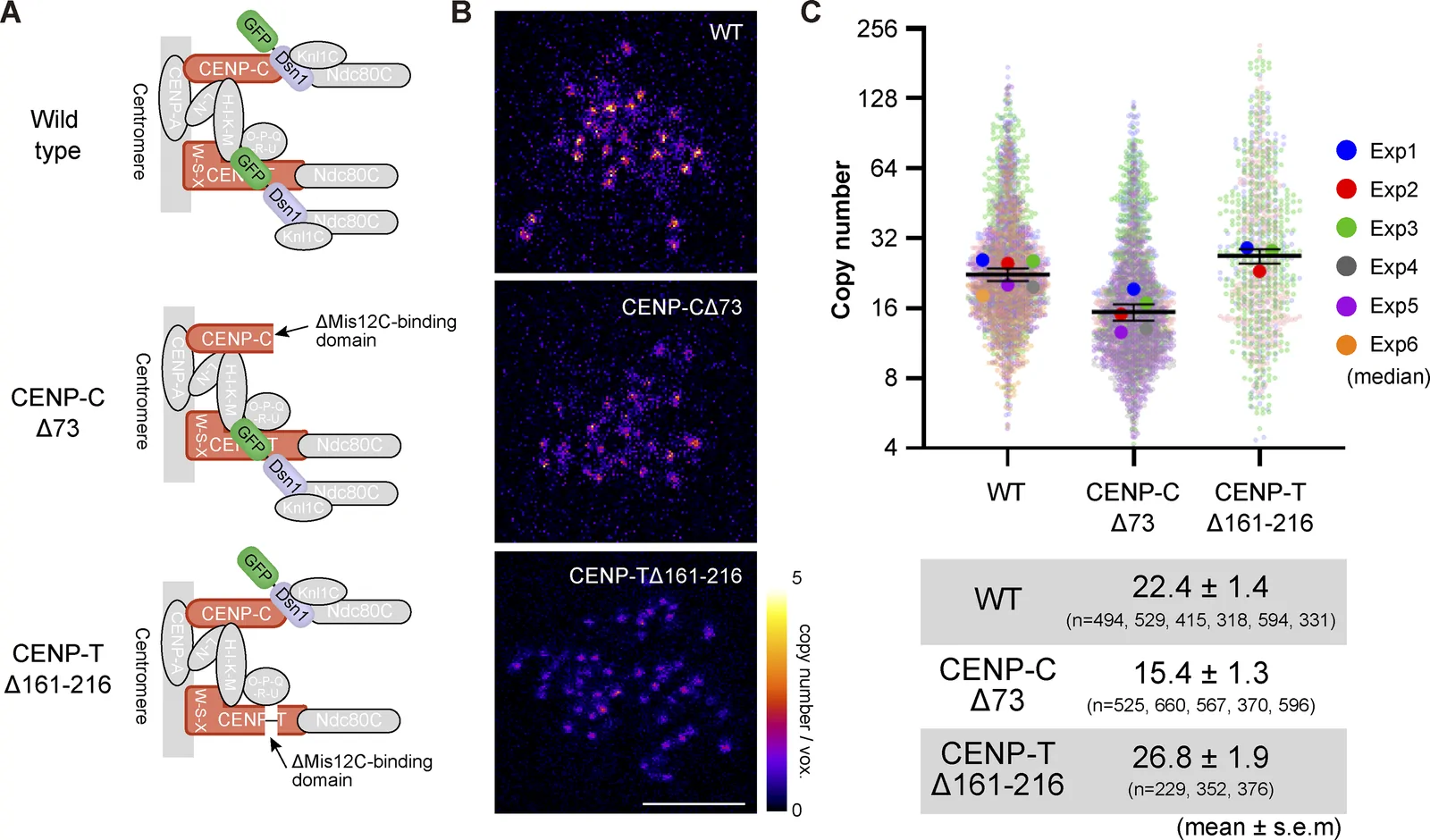
**The Mis12C is loaded onto kinetochore through CENP-T and -C redundant pathways. (A)** Schematic illustration of CENP-C– and CENP-T–mediated Mis12C loading pathways. The CENP-CΔ73 and CENP-TΔ161–216 strains express the mutant lacking the Mis12C-binding domain instead of WT CENP-C. **(B)** Representative copy number image of EGFP-Dsn1 in WT, CENP-CΔ73, and CENP-TΔ161–216 backgrounds. A color scale indicating protein copy number per voxel is shown on the right. Scale bar: 5 μm. **(C)** Copy number of Dsn1 molecules per kinetochore. The copy number of EGFP-Dsn1 in WT, CENP-CΔ73, and CENP-TΔ161–216 backgrounds was quantified from three to six independent experiments and is plotted as described in Fig. 1 C.

Next, we determined the copy number of the Mis12C in the KMN. We replaced the endogenous Dsn1 (a component of Mis12C) and CENP-T with EGFP- or mCherry-tagged protein, respectively (Fig. S3 A), and measured the Dsn1 copy number with FCS-calibrated counting. Dsn1 presents 22.4 ± 1.4 molecules at each mitotic kinetochore (Fig. 2, WT). Although Mis12C can interact with both CENP-C and CENP-T (Hara et al., 2018; Rago et al., 2015; Screpanti et al., 2011; Takenoshita et al., 2022; Takenoshita et al., 2024), the copy number of Dsn1 was lower than the combined copy number of CENP-C (∼26) and CENP-T (∼14) (Fig. 1 and Fig. 2 A). This result was consistent with previous studies showing that a subset of CENP-T or CENP-C molecules may not be engaged with Mis12C (Hara et al., 2018; Johnston et al., 2010).

We therefore sought to understand how Mis12C localizes on CENP-T or CENP-C at kinetochores. To address this point, we used CENP-CΔ73 cells in which endogenous CENP-C was replaced with the CENP-CΔ73 mutant lacking the Mis12C-binding site (Hara et al., 2018; Takenoshita et al., 2022; Takenoshita et al., 2024): CENP-C–binding Dsn1 molecules were removed from kinetochores in these cells (Fig. 2 A, CENP-CΔ73). We replaced Dsn1 and CENP-T with EGFP-Dsn1 and mCherry-CENP-T, respectively, in CENP-CΔ73 cells. This resulted in comparable expression levels with those in WT cells (Fig. S3, A and B). As shown in Fig. 2, B and C, the copy number of Dsn1 at each kinetochore was reduced from 22.4 to 15.4 ± 1.3; the level of CENP-T signal at the kinetochore was not changed in CENP-CΔ73 cells (Fig. S3 B). Based on these data, we infer that ∼15 Dsn1 molecules bind to ∼14 CENP-T, while only seven Dsn1 molecules bind to ∼26 CENP-C in WT cells. However, the actual number of Dsn1 molecules bound to CENP-T may be <15 because the loss of the Mis12C-binding site in CENP-C allows the slightly elevated cytosolic Mis12C concentration to enable CENP-T to recruit additional Dsn1 molecules. Thus, we next examined the number of Dsn1 molecules bound to CENP-C using CENP-TΔ161–216 cells lacking the Mis12C-binding site (Takenoshita et al., 2024; Fig. S2 A and Fig. S3 C). Contrary to expectations, this strain did not show detectable growth and mitotic defects (Fig. S3, D and E). As shown in Fig. 2, B and C, 26.8 ± 1.9 Dsn1 molecules were localized at kinetochores in CENP-TΔ161–216 cells. Although it was expected that seven Dsn1 molecules would localize at kinetochores in CENP-TΔ161–216 cells based on data in CENP-CΔ73 cells, the number remained unchanged compared with WT cells. As CENP-C is the sole binding site of Dsn1 in CENP-TΔ161–216 cells, most CENP-C molecules are likely engaged with Dsn1 in CENP-TΔ161–216 cells. These results suggest that CENP-T and CENP-C pathways for loading Mis12C onto kinetochores are redundant and that the ranges of Dsn1 molecules capable of binding to CENP-T or CENP-C are approximately 0–15 and 7–26, respectively (discussed later). Since the double mutation of CENP-CΔ73 and CENP-TΔ161–216 causes cell death (Takenoshita et al., 2022), maintaining at least ∼15 Mis12C molecules is required for functional kinetochore formation, regardless of which binding site is used.

### The number of Ndc80C at the kinetochore is maintained through redundant pathways via CENP-T and CENP-C

**Figure 3.**
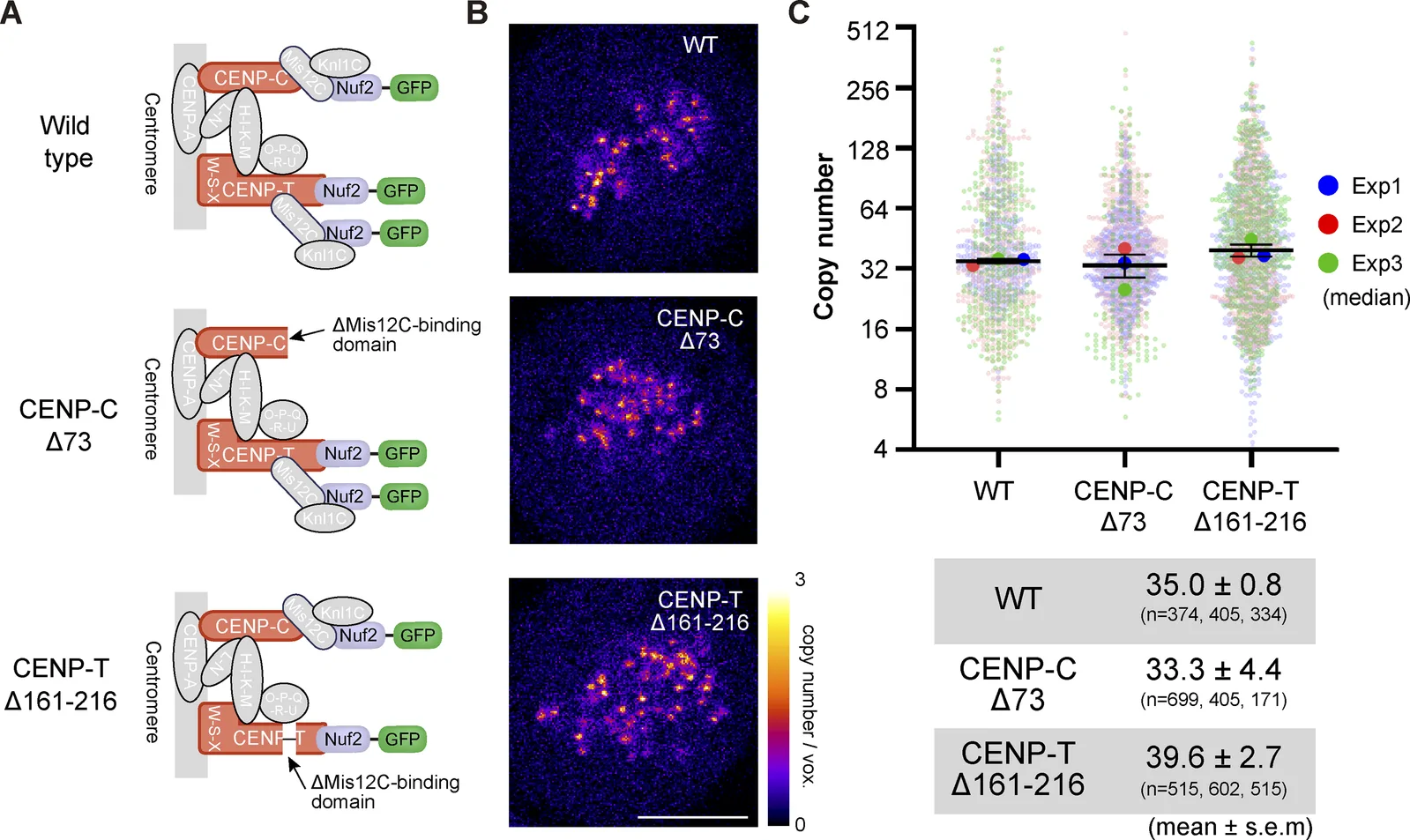
**The number of Ndc80C at the kinetochore is maintained through redundant pathways via CENP-T and CENP-C. (A)** Schematic illustration of CENP-C– and CENP-T–mediated Ndc80C loading pathways. **(B)** Representative copy number image of Nuf2-EGFP in WT, CENP-CΔ73, and CENP-TΔ161–216 backgrounds. A color scale indicating protein copy number per voxel is shown on the right. Scale bar: 5 μm. **(C)** Copy number of Nuf2 molecules per kinetochore. The copy number of Nuf2-EGFP in WT, CENP-CΔ73, and CENP-TΔ161–216 backgrounds was quantified from three independent experiments and is plotted as described in Fig. 1 C.

Next, we measured the number of Ndc80C molecules at each kinetochore in DT40 cells. Previous studies indicate that Ndc80C is recruited to kinetochore via three distinct sites in DT40 cells (Hara et al., 2018; Takenoshita et al., 2022): One directly binds to the CENP-T N terminus (aa 1–90 region) and another two bind to Mis12C on CENP-T (aa 161–216 region) or CENP-C (aa 1–73 region), thereby there are two Ndc80C-binding sites on CENP-T and one on CENP-C (Fig. 3 A). Because WT kinetochores contain 14 CENP-T, 26 CENP-C, and 22 Dsn1 molecules, biased localization of Ndc80C between CENP-C and CENP-T is likely. Under these constraints, the maximum number of Ndc80C molecules per kinetochore is predicted to be ∼36 (14 from CENP-T and 22 from Dsn1 on CENP-T and CENP-C).

**Figure S4.**
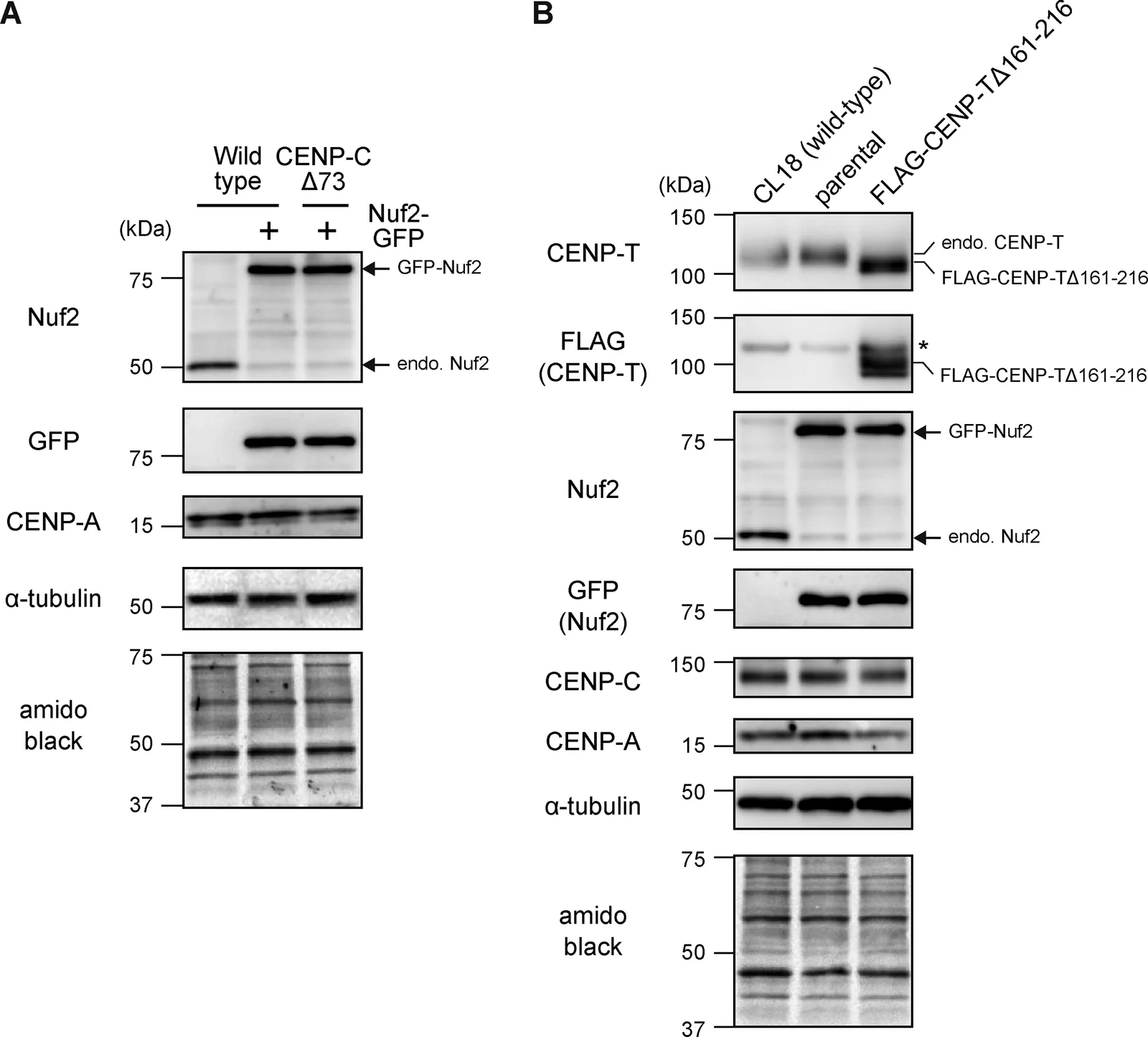
**Validation of cells used for the quantification of the copy number of Nudc80C. (A)** Protein levels of Nuf2-EGFP. Endogenous Nuf2 in WT and CENP-CΔ73 cells was replaced with Nuf2-EGFP. Protein levels of Nuf2, CENP-A, and α-tubulin were detected using antibodies indicated on the left. Protein molecular weight markers are indicated on the left. An amido black staining image for loading control is shown at the bottom. **(B)** Protein levels of FLAG-CENP-TΔ161–216. Endogenous CENP-T in CL18/Nuf2-EGFP was replaced with the FLAG-CENP-TΔ161–216 mutant. Protein levels of CENP-T, Nuf2, CENP-C, CENP-A, and α-tubulin were detected using antibodies indicated on the left. Protein molecular weight markers are indicated on the left. An amido black staining image for loading control is shown at the bottom. An asterisk on the right indicates a nonspecific band. Source data are available for this figure: SourceData FS4.

We replaced endogenous Nuf2 (a component of Ndc80C) with Nuf2-EGFP (Fig. S4 A) and quantified Nuf2 copy number by FCS-calibrated counting. We detected 35.0 ± 0.8 Nuf2 molecules per kinetochore in WT DT40 cells (Fig. 3, B and C), which is almost consistent with our prediction. Next, we analyzed how Ndc80C is loaded onto the kinetochore in CENP-CΔ73 and CENP-TΔ161–216 cells (Fig. S4). The Nuf2 copy number was unchanged in CENP-CΔ73 cells (33.3 ± 4.4) and showed a slight increase in CENP-TΔ161–216 cells (39.6 ± 2.7) (Fig. 3, B and C). In CENP-CΔ73 cells, the observed ratio of CENP-T, Dsn1, and Nuf2 numbers was ∼1:1:2, indicating that the two Ndc80C-binding sites on CENP-T are fully occupied (15 direct binding to CENP-T + 15 binding to Mis12C on CENP-T). In CENP-TΔ161–216 cells, the 26 Dsn1 molecules on CENP-C can bind to Ndc80C and the 14 CENP-T molecules can also be fully occupied by Nuf2, resulting in 39 Nuf2 was observed in these cells. Importantly, Mis12C is fully engaged with Nuf2 under all conditions tested. In addition, the direct Ndc80C-binding site on CENP-T is fully engaged with Nuf2 in both cases. Thus, the ranges of Nuf2 molecules capable of binding to CENP-T or CENP-C are ∼13–33 and 2–26, respectively (discussed later). These results suggest that maintaining more than 30 Ndc80C molecules by using CENP-T and CENP-C as redundant pathways is required for proper mitotic progression and cell growth (Fig. S3, D and E).

The previous observation has shown that ∼4.3 microtubules attach to each DT40 kinetochore (Ribeiro et al., 2009). This indicates that ∼8 Ndc80C attach to one microtubule in DT40 cells. In human cells, a single kinetochore contains 244 Ndc80C with ∼17.1 microtubules (Wendell et al., 1993), corresponding to ∼14 Ndc80C per microtubule (Suzuki et al., 2015). These observations indicate that a significant amount of Ndc80C is involved in establishing the kinetochore–microtubule attachment among vertebrates. Since clustering of Ndc80C stabilizes kinetochore–microtubule attachment (Polley et al., 2023), ∼10 Ndc80C appear to be required to generate a robust kinetochore–microtubule attachment.

### Mis12C and Ndc80C are more abundant in the cytosolic pool than at kinetochores

**Figure 4.**
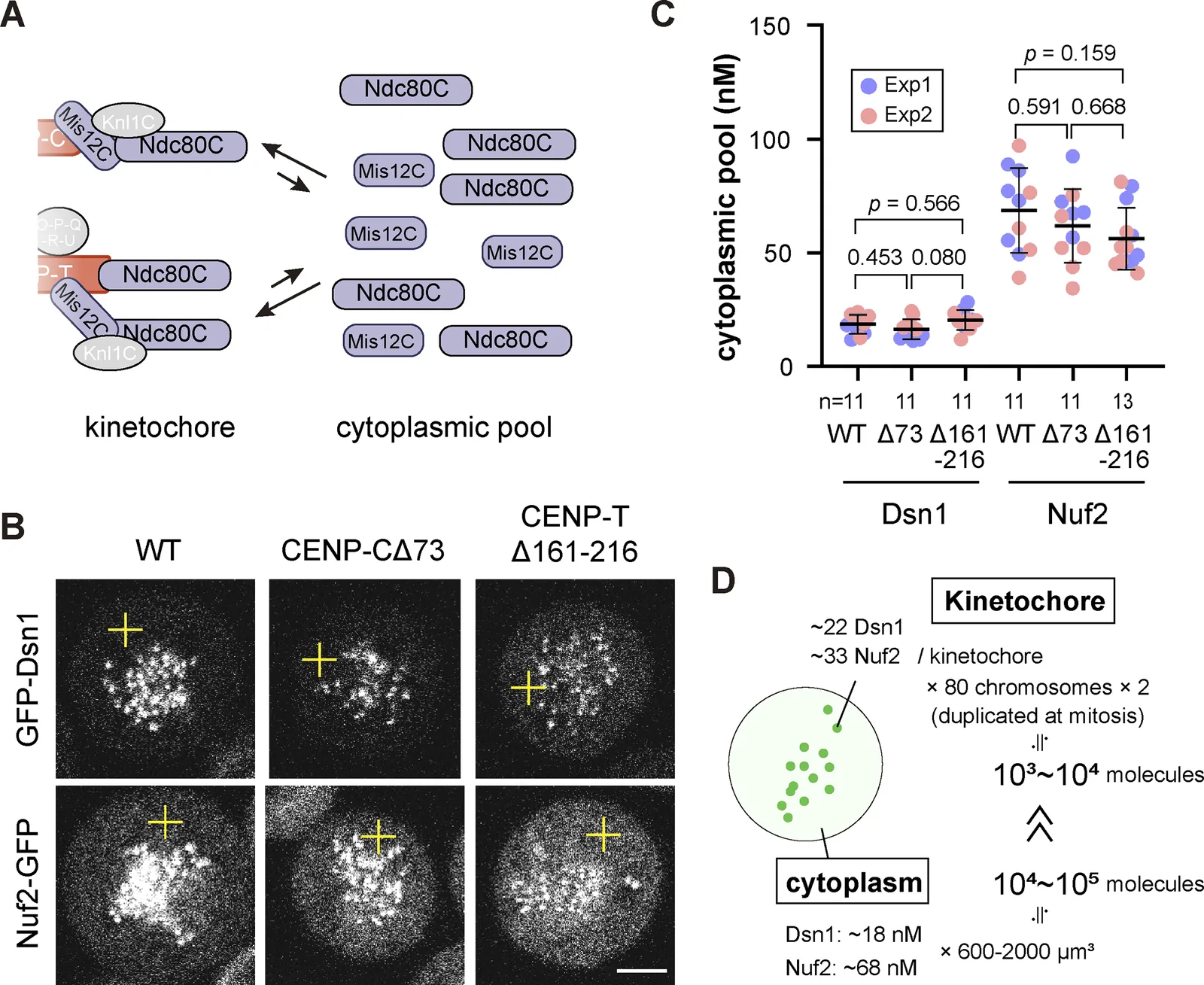
**Concentration of Dsn1 and Nuf2 in the cytosolic pools. (A)** Schematic illustration of molecular exchange between kinetochore and cytosolic fractions. **(B and C)** Concentrations of the cytosolic pools of Dsn1 and Nuf2. Representative confocal images showing the cytosolic pool of Dsn1 and Nuf2 in WT, CENP-CΔ73, and CENP-TΔ161–216 cells are presented in B. Yellow crosses indicate the positions of FCS measurements. Scale bar: 5 μm. Concentrations in the cytosolic pool determined by FCS are plotted in C. Horizontal lines in the plot represent the mean ± SD, calculated from 11 to 13 measurements across two independent experiments. Statistical significance was assessed using one-way ANOVA followed by Tukey’s multiple comparisons test. The p value is shown at the top. **(D)** Schematic of Dsn1 and Nuf2 molecules at kinetochores.

**Figure S5.**
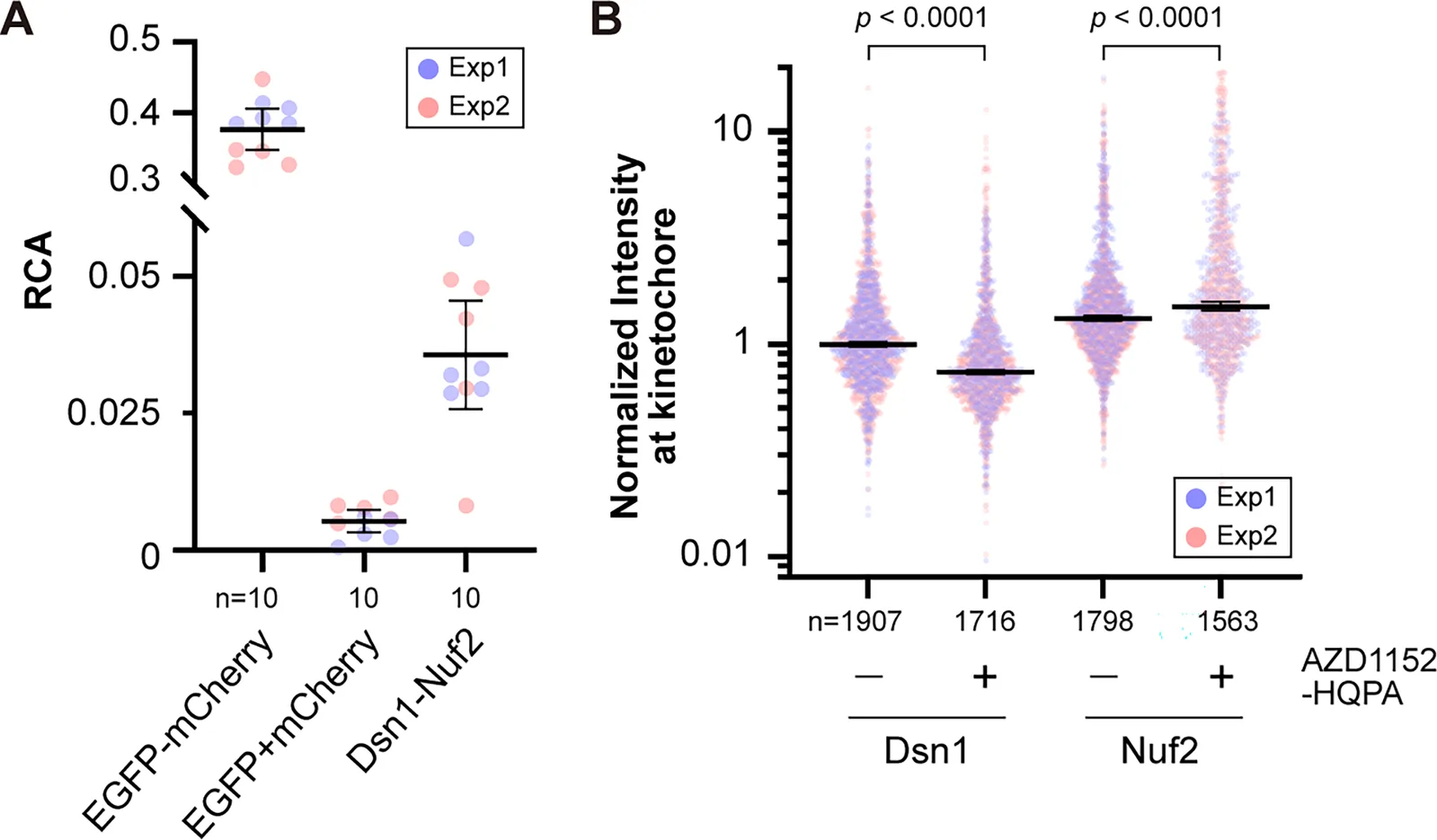
**FCCS measurement of Dsn1 and Nuf2 in mitotic cytoplasm and the level of Dsn1 and Nuf2 at kinetochores in the Aurora B inhibitor–treated cells. (A)** The amount of the Dsn1–Nuf2 complex in the mitotic cytoplasm was measured by FCCS. The RCA values of the EGFP-mCherry fusion protein (positive control), the individually expressed proteins (EGFP + mCherry, negative control), and the EGFP-Dsn1–Nuf2-mCherry from 10 cells across two independent experiments were plotted. Horizontal bars indicate the mean ± 95% CI. Statistical significance was assessed using a two-tailed Welch’s *t* test, and the p value is shown at the top. Data distribution was assumed to be normal, but this was not formally tested. The RCA of the Dsn1–Nuf2 complex was slightly above the negative control level, indicating that most Dsn1 and Nuf2 molecules do not interact with each other. **(B)** The levels of EGFP-Dsn1 and Nuf2-EGFP at mitotic kinetochores with or without AZD1152-HQPA treatment. DT40 cells expressing EGFP-Dsn1 and Nuf2-EGFP were treated with nocodazole for 3 h to arrest cells in metaphase. The cells were then released and treated with MG132 with (+) or without (−) AZD1152-HQPA. The normalized fluorescence intensity at the mitotic kinetochore was quantified across two independent experiments. Horizontal lines represent the median ± 95% CI of all measurements. The number of kinetochores analyzed from at least seven cells per experiment is shown at the bottom. Statistical significance was assessed using a two-tailed Welch’s *t* test, and the p value is shown at the top. Data distribution was assumed to be normal, but this was not formally tested.

The possible Mis12C-binding sites on CENP-T and CENP-C were completely occupied by Dsn1 in either CENP-TΔ161–216 or CENP-CΔ73 cells, suggesting that the binding-dissociation equilibrium is biased toward the kinetochore-bound state in cells. Thus, we compared the numbers of Mis12C and Ndc80C molecules in the kinetochore and cytosolic fractions to evaluate this equilibrium (Fig. 4 A). Using conventional FCS, we found that the concentrations of EGFP-Dsn1 and Nuf2-EGFP in WT cells were 18.6 ± 0.4 nM and 68.6 ± 18.6 nM, respectively, in the cytosolic pools (Fig. 4, B and C). Fluorescence cross-correlation spectroscopy (FCCS) revealed that most Dsn1 and Nuf2 molecules do not interact with each other in the cytoplasm (Fig. S5 A). Based on these concentrations and the measured volume of mitotic DT40 cells (636–2,208 µm^3^; *n* = 18), the numbers of Dsn1 and Nuf2 in the cytosolic pools are ∼10^4^–10^5^ molecules. At the kinetochore pool, the total numbers of Dsn1 and Nuf2 molecules at kinetochores in each cell are estimated to be ∼3,520 and ∼5,280, respectively: ∼22 Dsn1 and ∼33 Nuf2 molecules per kinetochore × ∼160 (∼80 chromosomes × 2) kinetochores per cell (Sonoda et al., 1998; Fig. 4 D). This estimation indicates that the numbers of these molecules at the kinetochore are one order of magnitude lower than those of the cytosolic pool (Fig. 4 D). Furthermore, the concentrations of Mis12C and Ndc80C in the cytosolic pools did not change between WT and mutant cells (Fig. 4, B and C). These results suggest that a large proportion of Mis12C and Ndc80C resides in the cytosolic pools and that the equilibrium is likely biased toward the kinetochore-bound state. Thus, the possible Mis12C-binding sites on CENP-T and CENP-C are completely occupied in CENP-TΔ161–216 or CENP-CΔ73 mutant cells. Conversely, in WT cells, once sufficient amounts of Mis12C and Ndc80C are loaded onto kinetochores, the remaining empty binding sites on either CENP-T or CENP-C appear to be inaccessible to additional KMN components.

### Mitotic kinetochore organization in DT40 cells

**Figure 5.**
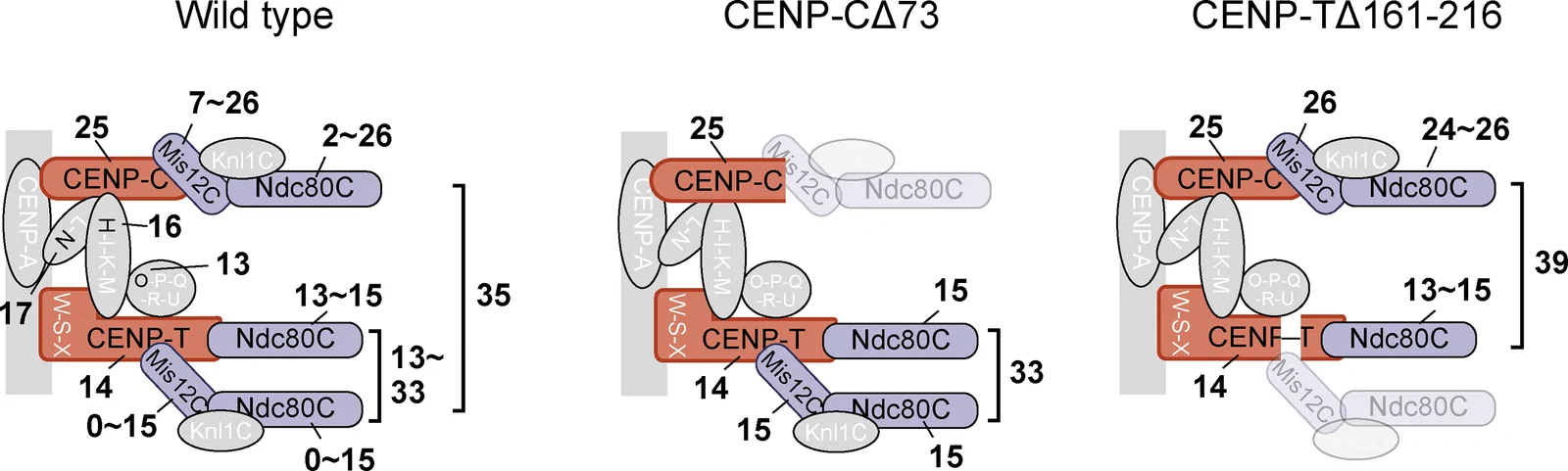
**A model of the kinetochore architecture including protein copy number at mitosis.** Protein copy-number ranges for kinetochore components per mitotic kinetochore were determined in WT, CENP-CΔ73, and CENP-TΔ161–216 cells. The stoichiometry of CCAN subcomplexes, except for CENP-C, is ∼1:1, whereas CENP-C is present at roughly twice the level of the other CCAN components. The direct binding of Ndc80C to CENP-T is likely the dominant contributor among KMN-loading sites. Mis12C is recruited through both CENP-T and CENP-C as redundant pathways, as long as the total number of Ndc80C molecules is maintained above ∼30 per kinetochore.

Based on these results, we propose a model of mitotic kinetochore architecture including copy numbers of each component in DT40 cells (Fig. 5): (1) ∼15 CCAN complexes assemble on the kinetochore, with equal stoichiometry among CCAN components except for CENP-C, which is present ∼26 copies at the kinetochore; (2) Mis12C and Ndc80C can engage with both CENP-T and CENP-C within certain molecular number ranges, and they can flexibly move on CENP-T and CENP-C; (3) for a functional kinetochore, total Ndc80C molecules must be maintained above 30.

Although Mis12C and Ndc80C can move on CENP-T and CENP-C, we propose that they preferentially bind to CENP-T in the WT with the following reasons: (1) CENP-TΔ90 cells lacking direct Ndc80C-binding site exhibits growth defects with abnormal mitotic behavior (Hara et al., 2018; Nishino et al., 2012; Takenoshita et al., 2022); (2) CENP-T-Mis12C–binding was largely reduced in CENP-TΔ90 cells, although a Mis12C-binding site in CENP-T still remained (Hara et al., 2018; Hori et al., 2013; Rago et al., 2015; Takenoshita et al., 2022), suggesting that direct binding of Ndc80C to CENP-T facilitates Mis12C–CENP-T interaction. If Mis12C and Ndc80C could efficiently bind to CENP-T in CENP-TΔ90 cells, they would be viable. However, this was not the case. Therefore, Ndc80C and Mis12C are likely preferentially loaded onto CENP-T rather than CENP-C. This biased localization of Mis12C on CENP-T was also observed in human cells (Suzuki et al., 2015). Currently, it is difficult to determine accurately how many Mis12C and Ndc80C complexes bind to CENP-T or CENP-C in WT cells, due to technical limitations. However, the number appears to reflect distinct binding mechanisms of Mis12C to CENP-T and CENP-C. Interactions between Mis12C and CENP-C or between Mis12C and CENP-T are facilitated by Mis12C phosphorylation by Aurora B kinase (Kim and Yu, 2015; Rago et al., 2015; Takenoshita et al., 2024). Indeed, the level of Dsn1 at kinetochores decreased with Aurora B inhibition by the specific inhibitor, AZD1152-HPQA, whereas the Nuf2 levels remained the same or slightly increased (Fig. S5 B). In addition to Mis12C phosphorylation, CENP-T phosphorylation also contributes to its Mis12C and Ndc80C interaction (Hara et al., 2018; Nishino et al., 2012; Takenoshita et al., 2024). This synergistic regulation might cause more efficient binding of Mis12C to CENP-T than CENP-C.

Ndc80C numbers (∼35 copies per kinetochore) on WT kinetochores were sustained in either CENP-TΔ161–216 or CENP-CΔ73 cells, suggesting that the number of Ndc80C is sufficient for generating a robust platform for stable microtubule attachment (8–10 Ndc80C per one microtubule). In addition, Ndc80C clusters via its loop domain stabilize end-on kinetochore–microtubule attachments under pulling forces from microtubules (Polley et al., 2023). We speculate that increasing the local copy number of Ndc80C promotes its clustering for stable kinetochore-spindle interactions—a phenomenon we refer to as the “platform effect” of clustered molecules. CCANs are also clustered at the kinetochore, and we propose that the CENP-C C terminus contributes to CCAN oligomerization through CENP-C–CCAN interactions, based on our previous studies (Ariyoshi and Fukagawa, 2023; Hara et al., 2023). Recently, another group and we independently showed that the CCAN forms a shell–core architecture, in which CENP-T and CENP-C localize to the outer “shell” and inner “core,” respectively (Gupta et al., 2026; Sakai et al., 2026). Because a substantial fraction of CENP-C is thought to reside within the core, KMN components may preferentially access CENP-T rather than CENP-C. Thus, a higher copy number of CENP-C relative to other CCAN components may be required to support this platform effect rather than its direct interaction with Mis12C. However, as most CENP-C binds to Mis12C in the CENP-TΔ161–216 cells, CENP-TΔ161–216 may alter the shell–core structure. The structural change likely generates a redundant pathway to maintain Ndc80C copy number at kinetochores.

In conclusion, this study reveals the theory-grounded copy number and stoichiometry of kinetochore proteins, which explains how kinetochore components are assembled and function.

## Materials and methods

### FCS-calibrated molecular number quantification to directly determine copy number of kinetochore proteins

To determine the copy number of kinetochore proteins within the chicken DT40 cells, we used the FCS-calibrated molecular number quantification method (Fig. 1 A). This approach converts the fluorescence intensity of confocal images into absolute molecular numbers using a calibration curve derived from FCS measurements of mobile fluorescent proteins. The HeLa cells expressing a mobile fluorescent protein used to tag the protein of interest were mixed with DT40 cells expressing an immobile or low-mobility protein such as a kinetochore protein tagged with a fluorescent protein: when we measured an EGFP-tagged kinetochore protein in DT40 cells, we prepared EGFP-expressing HeLa cells and mixed it. Then, the mixture was imaged by confocal microscopy. As FCS allows for the theoretical determination of the protein molecular number in cells expressing a mobile fluorescent protein, a calibration curve that links fluorescence intensity to molecular number can be established. This correlation is then used to convert the fluorescence intensity of the immobile or low-mobility proteins into their respective copy numbers. This approach overcomes the limitations of previous methods for quantifying protein copy number.

We first examined the linearity and reproducibility of this method. The HeLa cells expressing EGFP or mScarlet were mixed with DT40 cells expressing the EGFP- or mScarlet-fused kinetochore proteins, respectively (Fig. 1 A), and confocal images containing both DT40 and HeLa cells were acquired. Then, the number of standard fluorescent proteins in HeLa cells was determined by FCS. We note that DT40 cells were cultured at the same temperature as HeLa cells for over 2 days to standardize the molecular brightness of the EGFP because the molecular brightness of EGFP was temperature dependent (Park et al., 2024; Savchuk et al., 2019). Despite performing the FCS measurement at the same temperature, the molecular brightness of EGFP generated at 37°C and 38.5°C differed (Fig. S1 A). This result suggests that the variation arises from the heterogeneous maturation process of EGFP at different temperatures. Notably, this phenomenon was not observed in mScarlet (Fig. S1 A). Thus, we cultured both DT40 and HeLa cells at 37°C for at least 2 days before observation and then used them for FCS-calibrated quantification. 2 days is likely sufficient for replacing EGFP synthesized at 38.5°C with EGFP produced at 37°C, as our previous results indicate that most CENP-T protein turns over within ∼18 h (Sridhar et al., 2026, *Preprint*). Acquiring confocal images and performing FCS measurements within the same field and focal plane minimizes optical aberrations during measurement, thereby improving the accuracy of determining protein copy number. Fluorescence intensity from confocal images and protein copy number determined by FCS were plotted, and a conversion factor (CF) for translating fluorescence intensity into protein copy number was calculated by linear regression (Fig. 1 B). During this process, we evaluated the signal-to-noise ratio (S/N) in the FCS measurements and confocal images to estimate the impact of background signals (Fig. S1 C). The count rate for the case with the lowest copy number (Fig. S1 B, arrow) was 77.5 kHz, whereas the background count rate measured from HeLa cells not expressing EGFP was approximately 2–3 kHz. This yielded a S/N >25 during the FCS measurement. This high S/N ratio suggests that background fluorescence does not substantially influence the accuracy of copy-number determination. In the confocal images, the fluorescence intensity at the FCS-measured point was 1.6 times higher than the background intensity (2,165.7 vs. 1,352.5), which also indicates a sufficiently high S/N ratio. Since the kinetochore signals were consistently within the range of the FCS-measured points, the background in the confocal imaging is also unlikely to affect the accuracy of the copy number determination. However, we note that the S/N ratio in a confocal image may have a greater impact when quantifying proteins present at very low copy numbers. Across six independent experiments with EGFP, the linear regression yielded an R^2^ value of 0.9711 (Fig. S1 B), demonstrating the robustness and reproducibility of this method.

We selected HeLa cells instead of DT40 cells for generating the standard curve for the following two reasons: (1) Ease of transfection. For this experiment, we needed to express a fluorescent protein at various levels to generate a standard curve. Transient expression is well suited for this purpose. HeLa cells are more convenient for transient expression than DT40 cells because they are adherent cells. (2) Cell size. To accurately determine the molecular number, FCS must be performed in regions where the fluorescent protein is uniformly diffused, and no subcellular structures are present. Since HeLa cells are larger than DT40 cells, suitable positions for FCS measurements are easier to select. However, differences in cellular environments could potentially alter the brightness of EGFP molecules and affect the standard curve. To examine this possibility, we expressed EGFP in both cell types and compared the slopes of the resulting standard curves. Since the standard curves were nearly identical in both cell types (Fig. S1 D), we concluded that cell type does not affect the standard curve and that HeLa cells are suitable for generating a standard curve.

This method determines protein copy number independently of fluorophore color. Since red fluorescence proteins exhibit relatively weaker photostability than that of the green fluorescence proteins, we applied the normalization of the photobleaching (detrending) to ensure the measurement accuracy (Fig. S1 D). In addition, differences in the maturation kinetics of fluorescent proteins can lead to discrepancies in measured copy numbers, even when quantifying the same protein tagged with different fluorescent proteins. Thus, we examined whether this method yields the same molecular numbers in the green and red channels. To test this, we expressed an mScarlet-fused EGFP protein (mScarlet-EGFP) in HeLa cells and measured the copy number of the protein per unit volume in both channels by FCS (Fig. S1 E). The EGFP copy number was 1.2 times higher than the mScarlet copy number, suggesting that a small fraction of mScarlet does not emit fluorescence due to its poorer maturation kinetics compared with EGFP. Therefore, this difference must be considered when comparing copy numbers of green and red fluorescent proteins. Since FCS provides a theoretical basis for determining absolute numbers, this method offers quantification of the number of kinetochore proteins within cells without estimation.

### Cell culture

Chicken DT40 cells were cultured in DMEM (Nacalai Tesque) containing 10% FBS (Sigma-Aldrich) and 1% chicken serum (Thermo Fisher Scientific) at 38.5°C with 5% CO_2_. HeLa Kyoto cells (hereafter, HeLa cells; gift from Dr. Shuh Narumiya, Kyoto University, Kyoto, Japan) were cultured in DMEM containing 10% FBS at 37°C with 5% CO_2_. All cell lines utilized in this study are listed in Table S1.

### Plasmid constructions

All plasmid constructions were performed using In-Fusion HD (TAKARA) according to the manufacturer’s protocol. For mCherry-CENP-T expression, mCherry and *Gallus gallus* (gg) CENP-T cDNA sequences were amplified by PCR, purified, and then cloned into the plasmid containing ∼2-kb homology arms around the start codon of each gene with the HisD gene cassette and neomycin resistance gene cassette (pYH77 and pYH78). For mScarlet-AID-CENP-O expression, ggCENP-O cDNA sequences were amplified by PCR, purified, and then cloned downstream of the IRES of the plasmid OsTIR1-T2A-BSR_IRES containing ∼2-kb homology arms around the start codon of the β-actin (ACTB) gene. For Nuf2-mCherry expression, ggNuf2 cDNA sequences were amplified by PCR, purified, and then cloned into pmCherry-N1 (Clontech). For mScarlet-EGFP expression, the DNA sequence of EGFP was amplified by PCR, purified, and then cloned into pmScarlet-C1. pmScarlet_C1 was a gift from Dorus Gadella (Addgene plasmid # 85042; http://n2t.net/addgene:85042; RRID:Addgene_85042) (Bindels et al., 2017). Plasmid nomenclature employed in this study and oligo DNA information are summarized in Table S2.

### Generation of cell lines

To express the mCherry-CENP-T constructs under the control of the endogenous CENP-T promoter, plasmids pYH77 and pYH78 were transfected with pYT334 using the Neon Transfection System (Thermo Fisher Scientific) with six times of pulse (1,400 V, 5 ms) in CL18 (WT DT40 clone) and CENP-CΔ73 cells expressing EGFP-Dsn1 (Takenoshita et al., 2024). To express the Nuf2-EGFP constructs under the control of the endogenous Nuf2 promoter, plasmids pBSKS_Nuf2 KI_EGFP-Nuf2_PGK Puro^R^ and pBSKS_Nuf2 KI_EGFP-Nuf2_PGK EcoGPT were transfected with pX330_ggNuf2 as described above in CL18 cells. To endogenously replace CENP-T with CENP-TΔ161–216, plasmids pYT331 and pYT448 (Takenoshita et al., 2024) were transfected with pYT334 using the Neon Transfection System with six times of pulse (1,400 V, 5 ms) in CL18-expressing EGFP-Dsn1 or Nuf2-EGFP. To express mScarlet-AID-CENP-O, the plasmid containing mScarlet-AID-CENP-O was transfected with pX335_ggACTB containing sgRNA against a genomic sequence around the start codon of ACTB and SpCas9 nickase (D10A) gene, using Neon Transfection System with six times of pulse (1,400 V, 5 ms) in CENP-O knockout cells (Hori et al., 2008). The transfected cells were selected in the medium containing appropriate selection drugs (0.5 μg/ml puromycin [cat. no.: Z1305N; TAKARA] for the selection of Puro^R^ expression, 25 μg/ml mycophenolic acid [cat. no.: 138-11003; Fujifilm Wako] and 125 μg/ml xanthine [cat. no. X0626; Sigma-Aldrich] for the selection of EcoGPT expression, 25 μg/ml Blasticidin S hydrochloride [cat no.:029-18701; Wako] for the selection of BS^R^ expression, 2 mg/ml G418 [cat. no.: SC-290658; Santa Cruz Biotech] for the selection of Neo^R^ expression, and 25 μg/ml L-histidinol [cat. no.: H6647; Sigma-Aldrich] for the selection of HisD expression) in 96-well plates to isolate single colonies.

### Western blotting

The DT40 cells were counted, washed once with phosphate-buffered saline, and subsequently resuspended in resuspension buffer (50 mM Tris-HCl [pH8.0] and 1% SDS). After sonication, the protein concentration was determined by BCA assay (cat. no.: 10678484; Thermo Fisher Scientific). The protein extract (5 μg per sample) was subjected to SDS-PAGE (SuperSep Ace, 5–20%, cat. no.: 197-15011; Fujifilm Wako), and subsequently transferred to an Immobilon-P PVDF membrane (cat. no.: IPVH00010; Merck Millipore). After blocking the membrane using Blocking One (cat. no.: 03953-95; Nacalai Tesque) for 30 min at room temperature (25–28°C), the target protein was probed with primary antibodies as follow: anti-GFP antibody (1:5,000 dilution, Code 598, Lot 084; MBL), anti-RFP antibody (1:5,000 dilution, Code PM005, Lot 048; MBL), anti-FLAG M2 antibody (1:5,000 dilution, F1804; Sigma-Aldrich), anti-chicken CENP-T antibody (1:5,000 dilution [Hori et al., 2008]), anti-chicken Nuf2 antibody (1:5,000 dilution [Hori et al., 2003]), anti-chicken Dsn1 (1:5,000 dilution [Hara et al., 2018]), anti-chicken CENP-C (1:20,000 dilution, [Takenoshita et al., 2022]), anti-chicken CENP-A (1:5,000 dilution [Hori et al., 2020]) and anti-α-tubulin antibody (0.1 μg/ml, DM1A, AB_1965960; Thermo Fisher Scientific), followed by secondary antibodies: anti-rabbit IgG antibody labeled with HRP and anti-mouse IgG antibody labeled with HRP (111-035-144 and 315-035-003; Jakson laboratory). The bands were detected using ImmunoStar Zeta or LD (cat. nos.: 297-72403 and 292-69903; Fujifilm Wako, respectively) depending on their band intensity.

### FCS measurement

FCS measurement was performed using either LSM780 (for Figs. 2, 3, and 4) or LSM880 (for Fig. 1) (Carl Zeiss) equipped with 40× C-Apochromat/1.2NA W. UV-VIS-IR or 63× C-Apo/1.2 NA W. UV-VIS-IR water-immersion objective lens, respectively, at 25°C. The microscopes were driven by built-in software ZEN 2.3 SP1. The photons were recorded for 100 s. Count per molecule was set to approximately 1–3 kHz to reduce photobleaching during measurement, especially in the case of red fluorescent protein. Curve fitting analysis of the ACF was performed using ZEN software with a model for two-component 3D diffusion involving a one-component exponential blinking fraction, as shown in the following equation:

$$G\left(\tau \right)=1+G_{d}\left(\tau \right)\cdot G_{t}\left(\tau \right),$$

where $G_{d}\left(\tau \right)$and $G_{t}\left(\tau \right)$ represent the diffusion and exponential blinking fraction, respectively. $G_{d}\left(\tau \right)$ and $G_{t}\left(\tau \right)$ are expressed as follows

$$G_{d}\left(\tau \right)=\frac{1}{N}\cdot \overset{2}{\underset{i=1}{\sum }}\frac{F_{i}}{\left(1+\frac{\tau }{\tau_{d,i}}\right)\cdot \left(\sqrt{1+\frac{1}{S^{2}}\cdot \frac{\tau }{\tau_{d,i}}}\right)}$$

Gtτ=1+Tt1−Tt·e−ττt,

where N represents the average molecular number in the confocal detection volume during the measurement; S represents the structural parameter determined by analyzing the standard fluorescent dyes (Alexa 488 and Alexa 594) on the same day; $\tau_{d,i}$ represents the diffusion correlation time (i = 1 or 2); *F*_*i*_ represents the fraction of the component (i = 1 or 2); $T_{t}$, and $\tau_{t}$, represent the exponential blinking (also termed as fluorescence intermittency or triplet) faction and its meantime, respectively. In the case of the fitting analysis for red fluorescence protein, we normalized the photobleaching according to the following equation:

$$G\left(\tau \right)=1+G_{d}\left(\tau \right)\cdot G_{t}\left(\tau \right)\cdot G_{e}\left(\tau \right),$$

where $G_{e}\left(\tau \right)$ represents the stretched exponential. $G_{e}\left(\tau \right)$ is expressed as follows

Geτ=1−K+K· e−ττk,

where K represents the amplitude; $\tau_{k}$ represents the relaxation time.

Confocal volume ($V_{conf}$) for each experiment was determined by analyzing the standard fluorescent dyes. The $V_{conf}$ was calculated using the following equation:

Vconf=4π·D·τ32·S,

where D and τ represent the diffusion coefficient and the diffusion correlation time of the standard dye, respectively. Diffusion coefficient of Alexa 488 (430 μm^2^/s) and Alexa 594 (370 μm^2^/s) was referred to the value previously reported (Weber et al., 2004). When photobleaching was prominent during measurement, particularly for mScarlet, we applied detrending prior to ACF fitting. Fluorescence-intensity fluctuation data exhibiting photobleaching were fitted with a single-exponential decay and subsequently normalized using the fitted parameters. Cell volume of mitotic DT40 cells was measured using the Surface function of Imaris software (version 9.2.1, Oxford Instruments).

### FCS-calibrated protein copy number quantification

EGFP-C1 (Clontech) or mScarlet-C1 vector was transfected into HeLa cells using Lipofectamine 2000 or 3000 (Cat Nos. 11668027 and L3000008; Thermo Fisher Scientific) according to the manufacturer’s protocol, and the cells were incubated overnight at 37°C with 5% CO_2_. For FCS measurements, DT40 cells expressing each fluorescently labelled kinetochore protein were cultured at 37°C with 5% CO_2_ for at least 2 days prior to the experiment to ensure maturation of the fluorescent proteins in both HeLa and DT40 cells under identical temperature conditions. To synchronize cells at metaphase, DT40 cells expressing fluorescently labeled kinetochore protein were treated with 100 ng/ml nocodazole (cat. no.: M1404; Sigma-Aldrich) for 3 h. After washing the cells three times with medium, the cells were subsequently treated with 10 μM MG132 (cat. no.: C2211; Sigma-Aldrich) and 1 μM of SiR-DNA (cat no.: CY-SC007; Cytoskeleton, Inc.) or SPY650-DNA (cat no.: CY-SC501; Cytoskeleton, Inc.) in a 1:1,000 dilution for 30 min. The transfected HeLa cells were trypsinized, the cell number was counted using a Countess II (Thermo Fisher Scientific), mixed with the DT40 cells at a 1:5 ratio, and then attached to a Concanavalin A–coated 8-well chamber slide (cat. no.: 155411; Labtek). During the experiment, the cells were maintained in phenol red-free DMEM supplemented with 10% FBS, 1% chicken serum, 25 mM Hepes-NaOH (pH 7.4), and 10 μM MG132 at 25°C.

For quantification, a z-stack confocal image was initially acquired that included DT40 cells expressing the target kinetochore protein and HeLa cells transiently expressing the corresponding fluorescent protein (i.e., EGFP or mScarlet). Basically, we acquired confocal images that satisfied the Nyquist sampling theorem in all directions. When the fluorescent protein was prone to photobleaching, such as mCherry, we increased the z-step size to minimize photobleaching during image acquisition. FCS measurements were then conducted on the HeLa cells. An average fluorescence protein copy number in the confocal detection volume, N, was determined by fitting the ACF as described above. The average fluorescence intensity at the FCS measurement location was quantified using Fiji-ImageJ software (Schindelin et al., 2012): because the confocal volume spans roughly six pixels laterally in the image, we calculated the mean intensity within a circular region of six-pixel diameter. This intensity was subsequently plotted against the protein copy number, N, determined by FCS. The CF used to translate fluorescence intensity of kinetochore protein into protein copy number was obtained by linear regression using GraphPad Prism software (version 10.4 or higher).

Fluorescence intensity of the confocal image was converted to protein copy number per voxel ($N_{i,j,k}$) using the following equation:

$$N_{i,j,k}=\frac{F_{i,j,k}-F_{back}}{CF\cdot \left(V_{conf}/V_{voxel}\right)},$$

where $F_{i,j,k}\;\text{and}\;F_{back}$ represent the fluorescence intensity of each voxel (i,j,and k denote the voxel position along x-, y-, and z-axes, respectively) and the background intensity of the confocal image, respectively: $F_{back}$ is the averaged fluorescence intensity in regions where cells are absent. $V_{voxel}$ represents the voxel volume of the confocal image. Finally, individual kinetochores were segmented using the Spot function in Imaris software (version 9.2.1 or later), and the copy number of kinetochore protein per kinetochore was determined by summing counts within each segmented region. Kinetochore protein copy numbers were quantified from at least five cells per experiment, with each experiment independently repeated three to six times. Median values from each experiment and the mean ± standard error across all experiments were plotted.

### FCCS

FCCS analysis was performed using LSM880 as described in the “FCS measurement” section. Fluorescence-intensity fluctuation data for the green and red channels were collected using a multiband pass dichroic mirror (MBS 488/594) and grating (489–569 nm and 596–694 nm for green and red channels, respectively). Optical settings were calibrated using Alexa488 and Alexa594 as a control. mCherry-Nuf2 was transiently expressed in DT40 cells expressing EGFP-Dsn1 using the Neon Nxt transfection system (Thermo Fisher Scientific). After a day after transfection, the cells were treated with 100 ng/ml nocodazole for 3 h to arrest cells in metaphase. After release from mitotic arrest, the cells were attached to a Concanavalin A–coated 8-well chamber slide and maintained in phenol red-free DMEM supplemented with 10% FBS, 1% chicken serum, 25 mM Hepes-NaOH (pH7.4), and 10 μM MG132. For positive and negative controls of the FCCS measurement, we transiently expressed EGFP-mCherry fusion protein or EGFP and mCherry in HeLa cells and then performed FCCS measurements. Relative cross-correlation amplitude (RCA) was calculated using the following equation:

$$RCA=\frac{N_{gr}}{N_{r}},$$

where $N_{\text{gr}}$ and $N_{r}$ represent the number of red molecules and interacting molecules, respectively.

### Quantification of fluorescence intensity

DT40 cells expressing EGFP-Dsn1 and mCherry-CENP-T in either WT or CENP-CΔ73 backgrounds were treated with 100 ng/ml nocodazole for 3 h to arrest cells in metaphase. For WT cells, Hoechst 33342 (cat no.: AMBH9A260690; Sigma-Aldrich) was added at 50 ng/ml throughout the incubation period of nocodazole. For CENP-CΔ73 cells, Hoechst 33342 was added during the final 10 min to achieve weak DNA staining to distinguish WT and CENP-CΔ73 cells in the following observation. After three washes with medium, cells were resuspended in medium containing 10 μM MG132. WT and CENP-CΔ73 cells were mixed at a 1:1 ratio and seeded onto glass-bottom dishes (cat no.: P35G-1.5-14-C; Mattek) coated with concanavalin A. Following removal of unattached cells by washing, the medium was replaced with phenol red-free medium containing 10 μM MG132 and 25 mM Hepes-NaOH (pH 7.4). Fluorescence images were acquired using a DeltaVision Elite system (GE Healthcare) equipped with a 60× UPlanXApo/NA1.42 objective lens (Evident) and a pcoEdge 4.2 sCMOS camera (Excelitas Technologies). The average fluorescence intensity of mCherry-CENP-T at kinetochores was quantified using Imaris software, and values from 10 cells were plotted. Cell volume was quantified using “Surface” function in Imaris software.

To quantify EGFP-Dsn1 and Nuf2-EGFP signals at kinetochore under Aurora B kinase inhibition condition, DT40 cells expressing EGFP-Dsn1 and Nuf2-EGFP were treated with 100 ng/ml nocodazole for 3 h to arrest cells in metaphase, released, and then treated with 10 μM MG132 with or without 1 μM AZD1152-HQPA (cat. no.: SML0268; Merck) for 1 h. Images were acquired using an LSM880 equipped with 63× C-Apo/1.2NA W. UV-VIS-IR water-immersion objective lens. Kinetochore signals of EGFP-Dsn1 or Nuf2-EGFP were quantified using the spot function of Imaris software.

### Statistical analysis

The significance of mCherry-CENP-T, EGFP-Dsn1, and Nuf2-EGFP fluorescence intensity was assessed using a two-tailed Welch’s *t* test. The significance of the concentrations of the cytoplasmic pools of Dsn1 and Nuf2 was evaluated by one-way ANOVA followed by Tukey’s multiple comparisons test. The significance of RCA values was assessed using a two-tailed Welch’s *t* test. Data distribution was assumed to be normal, but this was not formally tested. All significance tests were performed using GraphPad Prism software as described above.

### Online supplemental material

Supplementary material includes five figures, two tables, and source data (Figs. S1, S2, S3, S4, and S5; Tables S1 and S2; and Source Data FS2, FS3, and FS4). Fig. S1 contains the validity of FCS-calibrated kinetochore protein copy number quantification, related to Fig. 1. Fig. S2 contains validation of protein expression level of CCAN proteins, related to Fig. 1. Fig. S3 contains validation of cells used for the quantification of the copy number of Mis12C, related to Fig. 2. Fig. S4 contains validation of cells used for the quantification of the copy number of Ndc80C, related to Fig. 3. Fig. S5 contains FCCS measurement of Dsn1 and Nuf2 in mitotic cytoplasm and the level of Dsn1 and Nuf2 at kinetochores in the Aurora B inhibitor–treated cells. Tables S1 and S2 contain cell lines, plasmids, and oligo DNAs used in this study.

## Supplementary Material

Review History

Table S1 shows DT40 strains used in this study.

Table S2 shows plasmids used in this study.

SourceData FS2 is the source file for Fig. S2.

SourceData FS3 is the source file for Fig. S3.

SourceData FS4 is the source file for Fig. S4.

## Data Availability

All data from this article are available from the corresponding author upon request.
