# Supplementary material for: Quantitative measurements of kinetochore protein copy numbers via FCS-calibrated molecular counting
Source: J Cell Biol. 2026 Nov 11;226(1):e202603185. doi: 10.1083/jcb.202603185 (PMC13753873; doi:10.1083/jcb.202603185)
Supplement: Supplementary file 1 [file jcb_202603185_review_history.pdf]

JCB

Quantitative measurements of kinetochore protein copy numbers via FCS-calibrated molecular counting

Yasuhiro Hirano, Yusuke Takenoshita, Akira Kitamura, Toru Hirota, and Tatsuo Fukagawa

Corresponding Author(s): Tatsuo Fukagawa, Osaka University

Review Timeline:

Submission Date:	2026-03-27
Editorial Decision:	2026-05-19
Revision Received:	2026-08-27
Editorial Decision:	2026-10-07
Revision Received:	2026-10-09

Monitoring Editor: Arshad Desai

Scientific Editor: Dan Simon

Transaction Report:

(Note: With the exception of the correction of typographical or spelling errors that could be a source of ambiguity, letters and reports are not edited. The original formatting of letters and referee reports may not be reflected in this compilation.)

DOI: https://doi.org/10.1083/jcb.202603185

May 19, 2026

Re: JCB manuscript #202603185

Tatsuo Fukagawa
Osaka University

Dear Prof. Fukagawa,

Thank you for submitting your manuscript entitled "Fluorescence correlation spectroscopy-calibrated molecular counting reveals the kinetochore architecture in chicken DT40 cells." The manuscript was assessed by expert reviewers, whose comments are appended to this letter. We invite you to submit a revision if you can address the reviewers' key concerns, as outlined here.

The reviewers have raised a number of points that will need to be addressed in the revision. Of particular importance are the comments and questions about the FCS methodology and calibration procedure, which should all be thoroughly answered.

Editorially, given that calibrated FCS is an established method, we believe this work is best suited for the Report format and not as a Tool. We recommend condensing the presentation to 5 primary figures with associated supplements (we can accommodate extra supplementary material if necessary) and focusing on the advances in understanding kinetochore architecture in situ.

While you are revising your manuscript, please also attend to the following editorial points to help expedite the publication of your manuscript. Please direct any editorial questions to the journal office.

GENERAL GUIDELINES:
Text limits: Character count for a Reports is < 20,000, not including spaces. Count includes title page, abstract, introduction, the joint Results & Discussion, and acknowledgments. Count does not include materials and methods, figure legends, references, tables, or supplemental legends.

** Reports must have a single combined 'Results and Discussion' section. **

Figures: Reports may have up to 5 main text figures. To avoid delays in production, figures must be prepared according to the policies outlined in our Instructions to Authors, under Data Presentation, https://jcb.rupress.org/site/misc/ifora.xhtml. All figures in accepted manuscripts will be screened prior to publication.

IMPORTANT: It is JCB policy that if requested, original data images must be made available. Failure to provide original images upon request will result in unavoidable delays in publication. Please ensure that you have access to all original microscopy and blot data images before submitting your revision.

Supplemental information: Reports are generally allowed up to 3 supplemental figures and 10 videos. As noted above, in this case, we will be able to give you extra space if needed.

Statistical analysis: Error bars on graphic representations of numerical data must be clearly described in the figure legend. The number of independent data points (n) represented in a graph must be indicated in the legend. Please indicate whether 'n' refers to technical or biological replicates (i.e. number of analyzed cells, samples or animals, number of independent experiments). If independent experiments with multiple biological replicates have been performed, we recommend using distribution-reproducibility SuperPlots (please see Lord et al., JCB 2020) to better display the distribution of the entire dataset, and report statistics (such as means, error bars, and P values) that address the reproducibility of the findings.

Statistical methods should be explained in full in the materials and methods. For figures presenting pooled data the statistical measure should be defined in the figure legends. Please also be sure to indicate the statistical tests used in each of your experiments (both in the figure legend itself and in a separate methods section) as well as the parameters of the test (for example, if you ran a t-test, please indicate if it was one- or two-sided, etc.). Also, if you used parametric tests, please indicate if the data distribution was tested for normality (and if so, how). If not, you must state something to the effect that "Data distribution was assumed to be normal but this was not formally tested."

Please note that JCB now requires authors to submit Source Data used to generate figures containing gels and Western blots with all revised manuscripts. This Source Data consists of fully uncropped and unprocessed images for each gel/blot displayed in the main and supplemental figures. For assays performed using capillary electrophoresis and/or immunoassay-based detection, authors should instead provide the electropherogram graph(s) for each experiment, plotting fluorescence/chemiluminescence intensity vs. molecular weight/size. Please be sure to provide one Source Data file for each figure gels, blots, and/or capillary electrophoresis assays along with your revised manuscript files. File names for Source Data figures should be alphanumeric without any spaces or special characters (i.e., SourceDataF#, where F# refers to the associated

main figure number or SourceDataFS# for those associated with Supplementary figures). For traditional gels and blots, the lanes of the gels/blots should be labeled as they are in the associated figure, the place where cropping was applied should be marked (with a box), and molecular weight/size standards should be labeled wherever possible. For capillary electrophoresis assays, each trace in the graph should be color-coded and labeled to indicate which protein, gene, or sample is being measured (please try to avoid red/green combinations to accommodate our color-blind readers).

Source Data files will be made available to reviewers during evaluation of revised manuscripts and, if your paper is eventually published in JCB, the files will be directly linked to specific figures in the published article.

Source Data Figures should be provided as individual PDF files (one file per figure). Authors should endeavor to retain a minimum resolution of 300 dpi or pixels per inch. Please review our instructions for export from Photoshop, Illustrator, and PowerPoint here: https://rupress.org/jcb/pages/submission-guidelines#revised

The typical timeframe for revisions is three to four months. If you anticipate any difficulties in meeting this aforementioned revision time limit, please contact us and we can work with you to find an appropriate time frame for resubmission. Please note that papers are generally considered through only one revision cycle, so any revised manuscript will likely be either accepted or rejected.

When submitting the revision, please include a cover letter addressing the reviewers' comments point by point. Please also highlight all changes in the text of the manuscript.

We hope that the comments below will prove constructive as your work progresses. We would be happy to discuss them further once you've had a chance to consider the points raised in this letter.

Thank you for this interesting contribution to Journal of Cell Biology. You can contact us at the journal office with any questions at cellbio@rockefeller.edu.

Sincerely,

Arshad Desai, PhD
Monitoring Editor
Journal of Cell Biology

Dan Simon, PhD
Scientific Editor
Journal of Cell Biology

Reviewer #1 (Comments to the Authors (Required)):

In this study, Hirano and colleagues define the composition of the kinetochore in DT40 chicken cells by reporting the absolute copy numbers of key protein subunits. Their work supersedes prior work on this topic by the Salmon lab in two important aspects. First, they use correlative Fluorescence Correlation Spectroscopy and confocal microscopy to enable direct, internally consistent determination of absolute protein counts in the confocal volume. Second, the authors also use well-defined mutant proteins to delineate the contributions of two parallel pathways for outer kinetochore assembly. The reported copy numbers for kinetochore subunits are accurate and reliable. They constitute the definitive view of the subunit composition of the DT40 kinetochores and will inform ultra structural and functional studies of the kinetochore. The authors' delineation of the contributions of the two outer kinetochore assembly pathways also provides useful information and raises interesting questions about the regulation of the same. Because of these reasons, I think the manuscript is suitable for the JCB audience. I do have a couple of technical comments that the authors should address. I also want to suggest three experiments that will significantly deepen the study. My comments are as follows.

1. Overall, the experiments were very carefully designed and executed. However, I was puzzled by the authors' use of HeLa cells for FCS standards, because this adds an unnecessary source of potential error. If this was due to technical reasons related to the DT40 system, the authors should include a clarification.

2. Lines 190-202: The authors conclude here that the main contributor to the observed variance in their reported copy number is the size of the centromere/kinetochore and technical sources of error are negligible. I suspect that the small volume occupied by the kinetochore, say ~ 50-100 nm3, relative to the focal volume (an ellipsoid with 600 nm minor diameter) will contribute a sampling error. Its magnitude will depend on the step size along the Z direction (optical axis). I could not find the step size used in the study. If it is missing, the authors should include it. If this step size is too large compared to the kinetochore size, then the measurements will suffer from a sampling error: some of the kinetochores will fall outside the center of the focal volume and appear dimmer than they are, and the inferred number of molecules will also be lower than expected. The authors should

discuss this issue. Ideally, they should ascertain the effect of this sampling issue is minimal by acquiring data for one protein using different Z step sizes.

3. The data delineating the Ndc80 and Mis12 complex recruitment by CENP-T an CENP-C and the intra-cellular concentrations of the two complexes (at least the subunits that were measured) are very informative. First, the intracellular concentrations of the two complexes are ~ 10-fold higher than the Kd's measured for some of the interactions; e.g., CENP-T-Spc24/25 Kd is ~ 0.5 uM from the author's prior work. Thus, one can expect the kinetochore binding sites for these complexes to be typically saturated. Second, only a fraction of the CENP-C molecules at the centromere interface with outer kinetochore proteins, presumably because they are not accessible. However, this fraction increases in the CENP-T(161-216) mutant. This intriguing observation suggests that the organization of the centromere might change in this mutant to enable the previously hidden CENP-C sites. Alternatively, there is some feedback in the recruitment of Mis12 and Ndc80 by the two proteins. The authors should discuss these possibilities.

4. A key question, especially in the CENP-C and CENP-T mutants is whether attachment to the MT contributes to stabilizing kinetochore composition and structure, e.g., by enabling Ndc80 molecules to cluster and thus stabilize their binding to the inner kinetochore. A simple experiment to test this notion would be to compare intensity measurements for Ndc80 by the mutants with wild-type proteins. Only confocal measurements will suffice.

5. I want to suggest one experiment that can deepen our understanding of kinetochore assembly (at the authors' discretion - the results will only add to the data presented). It would be informative to perform Fluorescence Cross-Correlation Spectroscopy measurements to quantify the fraction of cytosolic Ndc80 and Mis12 molecules associated with one another. These data will reveal whether their assembly is stepwise and if it explains the weak ability of the CENP-T(delta73) mutant to bind Mis12.

6. Lines 82-83 - The authors state that the accuracy of the old approach is limited by "significant measurement errors". While I understand the issue with using an exogenous fluorescence standard to convert intensity values into protein counts, the fluorescence measurements were performed with high accuracy.

7. Lines 345-46 - The sentence "If both were flexibly moving" should be revised for clarity.

8. In lines 234-236: "However, the actual number of Dsn1 molecules bound to CENP-T may be less than 15, because 1) Dsn1 bound to CENP-C can redistribute to CENP-T in CENP-CΔ73 cells, or 2) Dsn1 in the non-kinetochore fraction may bind to CENP-T in CENP-CΔ73 cells." This can be confusing to the reader and can be simplified as-= - 'in the absence of the Mis12-binding site in CENP-C, the slightly higher cytosolic Mis12 concentration may allow Dsn1 to recruit more molecules".

9. The authors refer to the free molecules of Dsn1 and Nuf2 as the "non-kinetochore" fraction. It would be more appropriate to call these molecules "cytosolic" for clarity.

10. In figure 1, instead of the label "conversion factor XXX, R2 YYY", it would be better to write the actual values. Also, in figure 1B, the x label "determined" is misspelled. Same figure, in the label they have EGFP misspelled as EGPF.

11. In line 172, they have "due to special resolution limit of confocal microscopy", should be spatial.

12. In 477 they say exponential blinking faction

Reviewer #2 (Comments to the Authors (Required)):

Summary:
Kinetochores are megadalton-sized, multi-subunit complexes that assemble at centromeres to connect the chromosomes to the microtubule-based mitotic spindle. The molecular architecture of kinetochores, including the copy number, arrangement, and linkages between components is thought to define their function in cells. Here, the authors use a new technique, brightness-calibrated FCS, to count the copy numbers of kinetochore components at centromeric puncta in chicken DT40 cells. The authors corroborate a finding reported in earlier work: that CENP-C is present at higher copy numbers relative to other CCAN components. Additionally, they newly report that when either CENP-C or CENP-T lack their Mis12c binding domains, the remaining assembly pathway can recruit Mis12c to match WT levels. We consider the submission of this manuscript to JCB's tools appropriate given the focus on methods. However, aspects of the figures and details of the methods should be revised prior to publication.

Comments:
• The observation that CENP-C is elevated in copy number relative to other components was reported in earlier work by the corresponding author (Johnston et al. 2010). Though Johnston et al. calibrated single fluorophore brightness using intensity measurements alone, the result newly reported here is substantively similar. Johnston et al. also report this number for DT40's. It remains unclear if CENP-C copy number elevation is a new finding.

• Fluorophores, especially GFP, exhibit lognormal brightness distributions with long tails towards larger brightness. For emitter counts under 10-15, this can lead to large errors in the determined copy number for an individual copy number estimate. The authors should add an error estimate to their intensity vs. copy number prediction in figure 1A. This is especially important as many of the reported copy numbers in figure 2 have a full quartile of the data below 10 copies, where this error estimate would be important to consider. A section of the introduction, discussion, or methods should address concerns of application of this technique for low protein copy numbers.
• Figure 3 should have a graphical depiction of the fluorophore location (e.g. on Dsn1/CENP-T)
• The authors find that recruitment of Mis12 and Ndc80c to CENP-C/CENP-T are not just redundant, but compensatory when mutations of CENP-C/CENP-T are made. The supposition that oligomerization of outer KT components (e.g. via the Ndc80c loop) can compensate for a lack of CENP-C or CENP-T binding sites seems speculative. It would benefit the manuscript for the authors to explore this idea through mutants of the Ndc80c loop domains. Alternatively, it is possible the additional/compensatory recruitment of Ndc80c/mis12 in cells with mutant CENP-C/CENP-T is related to the AurB phosphorylation of Mis12 and CENP-T. Imaging cells containing either phosphomimetic mutants of Mis12/CENP-T or an AurB kinase inhibitor washed in after Noc release would strengthen the work.
• The authors frequently describe FCS-calibrate brightness as a more accurate method than intensity comparison alone. However, they do not compare the relative accuracy of the two techniques on their samples. Furthermore, the copy number errors newly reported here are often within two-fold of those found previously using a brightness calibration alone (Johnston et al. 2010). Given the similarity of the errors, we ask the authors to show or quantify in more detail how FCS-calibrated brightness offers accuracy over canonical comparative brightness methods.
• Calibration of brightness B (or CPM) in coplated cells eliminates sources of intensity measurement variation from the microscope, coverslips, and imaging settings. However, the estimation of brightness B in co-plated HeLa cells relies explicitly on the assumption that the cellular environments of DT40's and HeLa's are similar enough in pH, oxidative state, and ion concentration to have identical brightness per GFP. This may not be true even between cells of the same type. It would be better to perform this experiment coplating cells with identical lineage, and better yet to obtain fluorescence calibrations directly from the cell of interest. At a minimum, to address this concern, we ask the authors to obtain brightness calibration estimates for both HeLa and DT40 cells to ensure these match well.

Reviewer #3 (Comments to the Authors (Required)):

Hirano et al. used the capability of Fluorescence Correlation Spectroscopy to measure absolute concentrations and brightnesses to investigate the composition of the kinetochore with focus on the CCAN and KMN networks. Using endogenous fluorescent protein knock-ins they created cell lines whose kinetochores could be analyzed by a calibrated FCS approach to determine the dependence of a pixel's intensity to its absolute number of contained fluorescence molecules. They use this to determine the stoichiometry of the kinetochore and demonstrate that KMN constituent complexes can bind to CCAN through either CENP-C or CENP-T. The data overall is convincing but some clarifications are required.

Comments

Molecular brightness, and thus the FCS-calibrated approach, is a complex property. Molecualr brightness not only depends on the photophysical properties but also on the mobility of the particle, its relation to acquisition time, and the background. The authros should provide background measurements and explain how they were incorporated in the calibrations. And some explanations are necessary to indicate how intensity and FCS can be calibrated on a fast-moving fluorescent protein and then be used on an almost immobile or very slow-moving particle. What is the influence of the diffusion coefficient?

What is the basis of the temperature dependence of the fluorescent protein brightness?

A large fraction of fluorescent proteins is typically non-fluorescent, especially in the case of red fluorescent proteins. This is a dark fraction that cannot be easily accounted for in a brightness approach. How do the authors account for these. Would it be possible to use a protein labeled with either EGFP or mScarlet and demonstrate that they provide the same copy number?

How is the observation volume difference at different wavelength included in the evaluation. In principle, red fluorescent protein labeled proteins will be seen over a larger volume.

The kinetochore assembly depends on the affinities of the proteins for each other. As has been shown by Hoischen et al (PLoS One 2018), using FCCS, the dissociation constants of the CENP proteins are in the 0.1-1 uM range. What concentrations do the authors work at and are they in the saturated concentration range?

The authors culture cells after transfection for at least two days to ensure maturation. But cells continuously express proteins and thus there is always a non-matured fraction. Is the expression rate after 48 hrs already at zero? It might be good to show protein production to estimate whether 48 hrs is sufficient to sufficiently reduce non-matured proteins.

What is the relation of the xy-step size of the confocal to its PSF? Are neighboring pixels overlapping and is this taken a=into

account in the evaluation?
And how was the back ground in line 528 determined, and is FCS corrected for that?

Minor issues
Line 172: special -> spatial

Response to Reviewers
Hirano et al. (Ms. No. 202603185)

Reviewer #1

In this study, Hirano and colleagues define the composition of the kinetochore in DT40 chicken cells by reporting the absolute copy numbers of key protein subunits. Their work supersedes prior work on this topic by the Salmon lab in two important aspects. First, they use correlative Fluorescence Correlation Spectroscopy and confocal microscopy to enable direct, internally consistent determination of absolute protein counts in the confocal volume. Second, the authors also use well-defined mutant proteins to delineate the contributions of two parallel pathways for outer kinetochore assembly. The reported copy numbers for kinetochore subunits are accurate and reliable. They constitute the definitive view of the subunit composition of the DT40 kinetochores and will inform ultra structural and functional studies of the kinetochore. The authors' delineation of the contributions of the two outer kinetochore assembly pathways also provides useful information and raises interesting questions about the regulation of the same. Because of these reasons, I think the manuscript is suitable for the JCB audience. I do have a couple of technical comments that the authors should address. I also want to suggest three experiments that will significantly deepen the study. My comments are as follows.

We appreciate the positive evaluation of our work and believe that we have addressed all of the concerns indicated below.

1. Overall, the experiments were very carefully designed and executed. However, I was puzzled by the authors' use of HeLa cells for FCS standards, because this adds an unnecessary source of potential error. If this was due to technical reasons related to the DT40 system, the authors should include a clarification.

We selected HeLa cells to generate the standard curve for the following reasons:
1. Ease of transfection:
In this experiment, we needed to express a fluorescent protein over a broad range of expression levels to generate a standard curve, and transient expression is ideal for this purpose. HeLa cells are particularly convenient for transient expression because they are adherent cells.
2. Cell size:
For accurate determination the number of molecules, FCS must be performed in regions where the fluorescent protein is uniformly diffused, meaning that no subcellular structures are present. HeLa cells are larger than DT40 cells, making it easier to identify suitable positions for FCS measurements.

We also measured the standard curve in HeLa and DT40 cells. As shown in revised Supplementary Fig. 1D, the standard curves generated in HeLa and DT40 cells exhibited nearly identical slopes. This result indicates that HeLa cells are suitable for generating standard-curves.

These points are described in Method section (Page 15, lines 444-457) of the revised version.

2. Lines 190-202: The authors conclude here that the main contributor to the observed variance in their reported copy number is the size of the centromere/kinetochore and technical sources of error are negligible. I suspect that the small volume occupied by the kinetochore, say ~ 50-100 nm3, relative to the focal volume (an ellipsoid with 600 nm minor diameter) will contribute a sampling error. Its magnitude will depend on the

step size along the Z direction (optical axis). I could not find the step size used in the study. If it is missing, the authors should include it. If this step size is too large compared to the kinetochore size, then the measurements will suffer from a sampling error: some of the kinetochores will fall outside the center of the focal volume and appear dimmer than they are, and the inferred number of molecules will also be lower than expected. The authors should discuss this issue. Ideally, they should ascertain the effect of this sampling issue is minimal by acquiring data for one protein using different Z step sizes.

We appreciate the reviewer's comment. We described the Z-step size in the Methods section (Page 20, lines 610-614). Essentially, we used Nyquist's sampling rate for all directions, including the Z-direction. However, preventing photobleaching was most important factor in obtaining an accurate molecular count in this experiment. Thus, we adjusted the Z-step size depending on the photostability of the fluorescent protein. When using a protein with weak photostability, such as red fluorescent protein, we used a Z-step larger than the Nyquist's sampling rate.

3. The data delineating the Ndc80 and Mis12 complex recruitment by CENP-T an CENP-C and the intra-cellular concentrations of the two complexes (at least the subunits that were measured) are very informative. First, the intracellular concentrations of the two complexes are ~ 10-fold higher than the Kd's measured for some of the interactions; e.g., CENP-T-Spc24/25 Kd is ~ 0.5 uM from the author's prior work. Thus, one can expect the kinetochore binding sites for these complexes to be typically saturated. Second, only a fraction of the CENP-C molecules at the centromere interface with outer kinetochore proteins, presumably because they are not accessible. However, this fraction increases in the CENP-T(161-216) mutant. This intriguing observation suggests that the organization of the centromere might change in this mutant to enable the previously hidden CENP-C sites. Alternatively, there is some feedback in the recruitment of Mis12 and Ndc80 by the two proteins. The authors should discuss these possibilities.

We appreciate and fully agree with the reviewer's comment. Regarding the structural alteration of the kinetochore when CENP-TΔ161-216 is expressed, we discussed it in Page 12, lines 366-382. We also discussed the regulatory mechanism of Mis12C recruitment to CENP-T and CENP-C in Page 11, lines 340-346.

4. A key question, especially in the CENP-C and CENP-T mutants is whether attachment to the MT contributes to stabilizing kinetochore composition and structure, e.g., by enabling Ndc80 molecules to cluster and thus stabilize their binding to the inner kinetochore. A simple experiment to test this notion would be to compare intensity measurements for Ndc80 by the mutants with wild-type proteins. Only confocal measurements will suffice.

We have compared the number of Ndc80C proteins in CENP-C and CENP-T mutants with the number of Ndc80C proteins in wild-type cells. We showed these data in revised Fig.3.

5. I want to suggest one experiment that can deepen our understanding of kinetochore assembly (at the authors' discretion - the results will only add to the data presented). It would be informative to perform Fluorescence Cross-Correlation Spectroscopy measurements to quantify the fraction of cytosolic Ndc80 and Mis12 molecules associated with one another. These data will reveal whether their assembly is stepwise and if it explains the weak ability of the CENP-T(delta73) mutant to bind Mis12.

As suggested by the reviewer, we measured the cytosolic interaction between Dsn1 and Nuf2 using fluorescence cross-correlation spectroscopy (FCCS). The results are included in Supplementary Fig. S5A and Page 10, lines 292-296 of the revised version.

> *6. Lines 82-83 - The authors state that the accuracy of the old approach is limited by "significant measurement errors". While I understand the issue with using an exogenous fluorescence standard to convert intensity values into protein counts, the fluorescence measurements were performed with high accuracy.*

We appreciate the reviewer's comment. As the reviewer noted, our previous description was misleading. We would like to emphasize the problem of using an exogenous fluorescence standard to convert intensity values into protein counts. We have carefully revised the text and clarified our point (Page 4, lines 96-101, and Page 6, lines 160-163).

> *7. Lines 345-46 - The sentence "If both were flexibly moving" should be revised for clarity.*

In response to the reviewer's suggestion, we revised the sentence for clarity (Page 11, lines 323-335).

> *8. In lines 234-236: "However, the actual number of Dsn1 molecules bound to CENP-T may be less than 15, because 1) Dsn1 bound to CENP-C can redistribute to CENP-T in CENP-CΔ73 cells, or 2) Dsn1 in the non-kinetochore fraction may bind to CENP-T in CENP-CΔ73 cells." This can be confusing to the reader and can be simplified as-= - 'in the absence of the Mis12-binding site in CENP-C, the slightly higher cytosolic Mis12 concentration may allow Dsn1 to recruit more molecules".*

In response to the reviewer's suggestion, we revised the sentence for clarity (Page 8, lines 212-214).

> *9. The authors refer to the free molecules of Dsn1 and Nuf2 as the "non-kinetochore" fraction. It would be more appropriate to call these molecules "cytosolic" for clarity.*

In response to the reviewer's suggestion, we revised the sentence for clarity (Page 10, lines 282-307, and Page 25, lines 784-790).

> *10. In figure 1, instead of the label "conversion factor XXX, R2 YYY", it would be better to write the actual values. Also, in figure 1B, the x label "determined" is misspelled. Same figure, in the label they have EGFP misspelled as EGPF.*

Thank you for pointing out the ambiguous labeling and misspelling in Figure 1. We have changed the label in Figure 1 of the revised version. We have included the actual values of the conversion factor and R^2 value as the Reviewer suggested (see revised Figure 1).

> *11. In line 172, they have "due to special resolution limit of confocal microscopy", should be spatial..*

Thank you for pointing out the miswording. We have corrected it (Page 6, line 143).

> *12. In 477 they say exponential blinking faction*

Although we may not have fully understood the reviewer's point, we have added

additional terminology to clarify what the exponential blinking fraction represents and improve reader understanding (Pages 18-19, lines 568-569).

<u>Reviewer #2</u>

Summary

Kinetochores are megadalton-sized, multi-subunit complexes that assemble at centromeres to connect the chromosomes to the microtubule-based mitotic spindle. The molecular architecture of kinetochores, including the copy number, arrangement, and linkages between components is thought to define their function in cells. Here, the authors use a new technique, brightness-calibrated FCS, to count the copy numbers of kinetochore components at centromeric puncta in chicken DT40 cells. The authors corroborate a finding reported in earlier work: that CENP-C is present at higher copy numbers relative to other CCAN components. Additionally, they newly report that when either CENP-C or CENP-T lack their Mis12c binding domains, the remaining assembly pathway can recruit Mis12c to match WT levels. We consider the submission of this manuscript to JCB's tools appropriate given the focus on methods. However, aspects of the figures and details of the methods should be revised prior to publication.

We appreciate the positive evaluation from this Reviewer. We conducted additional experiments to strengthen our conclusion in response to comments from this Reviewer.

Comments:

• The observation that CENP-C is elevated in copy number relative to other components was reported in earlier work by the corresponding author (Johnston et al. 2010). Though Johnston et al. calibrated single fluorophore brightness using intensity measurements alone, the result newly reported here is substantively similar. Johnston et al. also report this number for DT40's. It remains unclear if CENP-C copy number elevation is a new finding.

As suggested by the reviewer, we have revised our conclusion regarding the CENP-C copy number (Page 2, line 40, Page 6, line 151, and Page 7, lines 178-181). We agree that the reason for the higher CENP-C copy number compared to other CCAN components remains unclear. However, we propose that a certain number of CENP-C is required to generate a functional kinetochore in interphase. Previously, we reported that the C-terminal cupin domain of CENP-C forms a multimer, and that the multimerization is essential for CENP-C function (Hara et al., Mol Cell, 2023). In addition, we recently demonstrated that this property is necessary for forming a spherical shell-like structure of CCAN units (Sakai et al, BioRxiv, 2026). These results suggest that a greater number of CENP-C are necessary to maintain the shell-like structure as a structural platform. We discuss this point in Page 12, lines 366-377 of the revised version.

• Fluorophores, especially GFP, exhibit lognormal brightness distributions with long tails towards larger brightness. For emitter counts under 10-15, this can lead to large errors in the determined copy number for an individual copy number estimate. The authors should add an error estimate to their intensity vs. copy number prediction in figure 1A. This is especially important as many of the reported copy numbers in figure 2 have a full quartile of the data below 10 copies, where this error estimate would be important to consider. A section of the introduction, discussion, or methods should address concerns of application of this technique for low protein copy numbers.

Thank you for pointing out this important issue. In the Method section of the revised version, we addressed the potential impact of background signals when

quantifying proteins present at low copy numbers. In our measurements, the fluorescence signals obtained during both FCS and confocal imaging were sufficiently high relative to the background (S/N values >25 for FCS and 1.6 for confocal imaging; see revised Supplementary Fig. 1C). Therefore, we concluded that background fluorescence was unlikely to compromise the accuracy of our copy-number determination. However, since this consideration is crucial for interpreting measurements of low-abundance proteins, we discussed it explicitly in the revised Method section (Page 15, lines 427-440).

> • *Figure 3 should have a graphical depiction of the fluorophore location (e.g. on Dsn1/CENP-T).*

Per the reviewer's suggestion, we have included graphical depictions of the fluorophore locations for Dsn1 (Fig. 3) and Nuf2 (Fig. 4).

> • *The authors find that recruitment of Mis12 and Ndc80c to CENP-C/CENP-T are not just redundant, but compensatory when mutations of CENP-C/CENP-T are made. The supposition that oligomerization of outer KT components (e.g. via the Ndc80c loop) can compensate for a lack of CENP-C or CENP-T binding sites seems speculative. It would benefit the manuscript for the authors to explore this idea through mutants of the Ndc80c loop domains. Alternatively, it is possible the additional/compensatory recruitment of Ndc80c/mis12 in cells with mutant CENP-C/CENP-T is related to the AurB phosphorylation of Mis12 and CENP-T. Imaging cells containing either phosphomimetic mutants of Mis12/CENP-T or an AurB kinase inhibitor washed in after Noc release would strengthen the work.*

Per the reviewer's suggestion, we have stated in the revised text (Page 12, lines 359–364) that our description of the role of the loop domain in Ndc80C is speculative. In addition, we quantified the kinetochore localization of Mis12C (Dsn1) and Ndc80C (Nuf2) in the presence of the Aurora B inhibitor AZD1152-HQPA. We demonstrated that Mis12C, but not Ndc80C, was reduced in this condition (see Supplementary Fig. 5B). These results support our model, and we described results in Page 11, lines 344-346 of the revised version.

> • *The authors frequently describe FCS-calibrate brightness as a more accurate method than intensity comparison alone. However, they do not compare the relative accuracy of the two techniques on their samples. Furthermore, the copy number errors newly reported here are often within two-fold of those found previously using a brightness calibration alone (Johnston et al. 2010). Given the similarity of the errors, we ask the authors to show or quantify in more detail how FCS-calibrated brightness offers accuracy over canonical comparative brightness methods.*

We appreciate the reviewer's comment and agree that our previous description was an overstatement and a misuse of words. As the reviewer pointed out, we would like to emphasize the problem that an exogenous fluorescence standard is used to convert intensity values into protein counts. We carefully revised our text, explained our point, and reworded some phrases (Page 4, lines 96-101, and Page 6, lines 160-163).

> • *Calibration of brightness B (or CPM) in coplated cells eliminates sources of intensity measurement variation from the microscope, coverslips, and imaging settings. However, the estimation of brightness B in co-plated HeLa cells relies explicitly on the assumption that the cellular environments of DT40's and HeLa's are similar enough in pH, oxidative state, and ion concentration to have identical brightness per GFP. This may not be true even between cells of the same type. It would be better to perform this experiment coplating cells with identical lineage, and better yet to obtain fluorescence*

calibrations directly from the cell of interest. At a minimum, to address this concern, we ask the authors to obtain brightness calibration estimates for both HeLa and DT40 cells to ensure these match well.

We agreed with the reviewer's suggestion, and compared the standard curves generated in HeLa and DT40 cells. The results are included as Supplementary Fig. 1D and are described in the Method section (Page 15, lines 452-457). Since the slopes were nearly identical, the EGFP brightness was same in HeLa and DT40 cells. In the revised text, we also mentioned the merits of using HeLa cells in our measurements (Page 15, lines 444-452). In conclusion, HeLa cells are suitable for generating the standard curves in our experiments.

<u>Reviewer #3</u>

Hirano et al. used the capability of Fluorescence Correlation Spectroscopy to measure absolute concentrations and brightnesses to investigate the composition of the kinetochore with focus on the CCAN and KMN networks. Using endogenous fluorescent protein knock-ins they created cell lines whose kinetochores could be analyzed by a calibrated FCS approach to determine the dependence of a pixel's intensity to its absolute number of contained fluorescence molecules. They use this to determine the stoichiometry of the kinetochore and demonstrate that KMN constituent complexes can bind to CCAN through either CENP-C or CENP-T. The data overall is convincing but some clarifications are required.

We appreciate positive evaluation. We try to address concerns from reviewers for clarity.

Comments
Molecular brightness, and thus the FCS-calibrated approach, is a complex property. Molecualr brightness not only depends on the photophysical properties but also on the mobility of the particle, its relation to acquisition time, and the background. The authors should provide background measurements and explain how they were incorporated in the calibrations. And some explanations are necessary to indicate how intensity and FCS can be calibrated on a fast-moving fluorescent protein and then be used on an almost immobile or very slow-moving particle. What is the influence of the diffusion coefficient?

We appreciate valuable suggestions. During the FCS measurements, we did not quantify the background signal within each cell because separating background fluorescence from cytosolic GFP on a cell-by-cell basis is difficult. Instead, we measured the background in non-transfected cells, which have no GFP signal. The background level was approximately 2–3 kHz, which is more than 25-fold lower than the weakest GFP signal detected during standard-curve generation (see revised Supplementary Fig. 1C). Such a low background level is unlikely to affect the accuracy of the standard curve. These points are described in the Method section (Page 15, lines 427-440) of the revised version.

Regarding the diffusion coefficient, it does not affect the number determined by FCS because the molecular number is calculated using the following equation:

$G(0) = 1 + 1/\langle N \rangle$

where G(0) is the y-axis intercept of the autocorrelation curve and <N> is the average molecular number during data acquisition. Since this equation does not contain any diffusion-related parameters, the diffusion coefficient does not influence the number determination.

In addition, diffusion does not affect fluorescence intensity during confocal imaging. Molecular diffusion is much faster than the scanning speed of confocal microscopy; therefore, the recorded intensity reflects the time-averaged number of fluorescent molecules within the focal volume.

> *What is the basis of the temperature dependence of the fluorescent protein brightness?*

The temperature dependency of EGFP fluorescence emission has been previously reported, e.g., in Savchuk OA et al. (*Sci. Rep.* (2019) DOI: https://doi.org/10.1038/s41598-019-44023-7) and Park W. et al. (*J. Phys. Chem. Lett.* (2024) DOI: https://doi.org/10.1021/acs.jpclett.4c02761). Although the chemical details of this process are beyond the scope of this study, we believe that the difference in molecular brightness at different temperature is informative for a broad reader of JCB. We cited the papers and revised the sentence in the Method section (Page 14, line 412).

> *A large fraction of fluorescent proteins is typically non-fluorescent, especially in the case of red fluorescent proteins. This is a dark fraction that cannot be easily accounted for in a brightness approach. How do the authors account for these. Would it be possible to use a protein labeled with either EGFP or mScarlet and demonstrate that they provide the same copy number?*

Recognizing the importance of this issue for our measurements, we evaluated the dark fraction of mScarlet. For this analysis, we transiently expressed an EGFP–mScarlet fusion protein and quantified the copy numbers of EGFP and mScarlet within the normalized confocal volume. The resulting data are presented in Supplementary Fig. 1F. We also described this in the Method section (Page 16, lines 462-472).

> *How is the observation volume difference at different wavelength included in the evaluation. In principle, red fluorescent protein labeled proteins will be seen over a larger volume.*

We agree that this is an important point. We converted the confocal volume measured by FCS to a voxel by dividing the confocal volume by the voxel size in confocal image. This normalized differences in confocal volume at different wavelengths. In other cases, we compared copy numbers in normalized volumes, such as per femtoliter. We replotted the data using these normalized values (see Fig.1A and Supplementary Figs 1B and 1D).

> *The kinetochore assembly depends on the affinities of the proteins for each other. As has been shown by Hoischen et al (PLoS One 2018), using FCCS, the dissociation constants of the CENP proteins are in the 0.1-1 uM range. What concentrations do the authors work at and are they in the saturated concentration range?*

We measured the concentrations of Dsn1 and Nuf2 in the cytoplasm and presented the data in Figs. 4B and 4C. The concentrations were 18.6 nM and 68.6 nM, which are lower concentration than the Kd range.

> *The authors culture cells after transfection for at least two days to ensure maturation. But cells continuously express proteins and thus there is always a non-matured fraction. Is the expression rate after 48 hrs already at zero? It might be good to show protein production to estimate whether 48 hrs is sufficient to sufficiently reduce non-matured proteins.*

We stably expressed fluorescently-tagged proteins, not transiently, so the cells

continuously express the target proteins. The most important aspect of this experiment is replacing fluorescent proteins—especially GFP that matured at 38.5 °C—with those matured at 37 °C. We previously reported that pulse-labeled CENP-T and CENP-S, components of CCAN, were almost undetectable within 18 hours by our SNAP-assay (Sridhar et al, BioRxiv, 2026). Here, we present the data from that study (see below). These data indicate that 48 hours is sufficient time to replace newly expressed proteins. We have added these descriptions in the revised Method section (Page 14, lines 417-421).

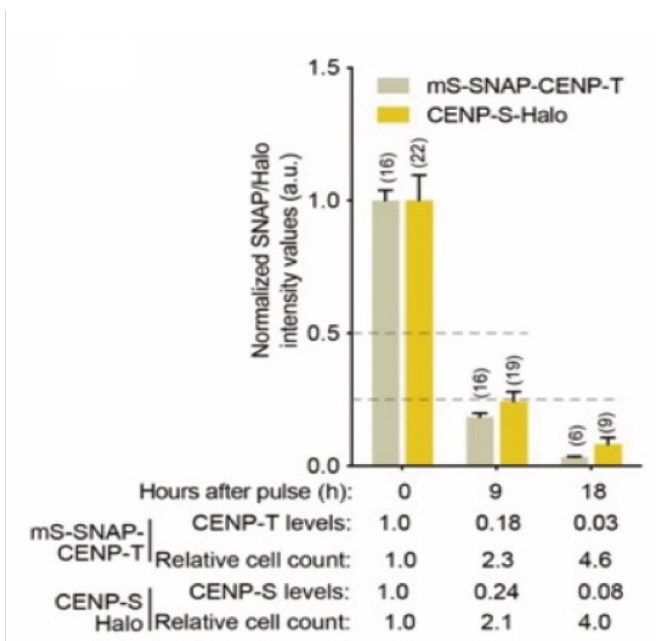

Fig. for the Reviewer
Localization of SNAP or Halo labelled CENP-T or CENP-S, respectively, over 0, 9 and 18 h of the pulse chase assay. SNAP or Halo labelled signal intensities in mitotic cells were quantified. (Data from Sridhar et al. BioRxiv, 2026)

> *What is the relation of the xy-step size of the confocal to its PSF? Are neighboring pixels overlapping and is this taken a=into account in the evaluation?*
> *And how was the background in line 528 determined, and is FCS corrected for that?)*

We appreciate the reviewer’s comment. We used Nyquist’s sampling rate for all directions. Thus, the pixel size for xy is more than half the size of its PSF. This point is described in the Materials and Methods section (Page 16, lines 511-515).

Regarding the overlapping problem, we attempted to eliminate overlapping kinetochores from the count by applying a stringent threshold during segmentation. However, it was impossible to completely remove them from the analysis. This is a technical limitation of the method. We explicitly explained this in the revised main text (Page 20, lines 610-614).

For confocal images, we measured the mean intensity where cells are absent and subtracted it as background. In contrast, we did not assess or subtract the background during FCS measurement because it is technically difficult to assess background (mainly derived from autofluorescence) in each cell. These points are explained in the Methods section (Page 20, Lines 629-630).

> *Minor issues*
> *Line 172: special -> spatial*

Thank you for pointing out the miswording. We have corrected it (Page 6, Line 143).

October 7, 2026

RE: JCB Manuscript #202603185R

Tatsuo Fukagawa
Osaka University

Dear Prof. Fukagawa,

Thank you for submitting your revised manuscript entitled "FCS-calibrated molecular counting reveals the kinetochore organization in DT40 cells." The manuscript has now been assessed by the three original reviewers. We would be happy to publish your paper in JCB pending final revisions necessary to meet our formatting guidelines (see details below).

To avoid unnecessary delays in the acceptance and publication of your paper, please read the following information carefully.

A. MANUSCRIPT ORGANIZATION AND FORMATTING:

Full guidelines are available on our Instructions for Authors page, https://jcb.rupress.org/submission-guidelines#revised.
Submission of a paper that does not conform to JCB guidelines will delay the acceptance of your manuscript.

1) Text limits: Character count for Reports is < 20,000, not including spaces. Count includes title page, abstract, introduction, results & discussion, and acknowledgments. Count does not include materials and methods, figure legends, references, tables, or supplemental legends.

2) Figure formatting: Reports may have up to 5 main text figures. Scale bars must be present on all microscopy images, including inset magnifications. Molecular weight or nucleic acid size markers must be included on all gel electrophoresis. Please add scale bars to figures 1A and S1C.

Also, please avoid pairing red and green for images and graphs to ensure legibility for color-blind readers. If red and green are paired for images, please ensure that the particular red and green hues used in micrographs are distinctive with any of the colorblind types. If not, please modify colors accordingly or provide separate images of the individual channels.

3) Statistical analysis: Error bars on graphic representations of numerical data must be clearly described in the figure legend. The number of independent data points (n) represented in a graph must be indicated in the legend. Please indicate whether 'n' refers to technical or biological replicates (i.e. number of analyzed cells, samples or animals, number of independent experiments). If independent experiments with multiple biological replicates have been performed, we recommend using distribution-reproducibility SuperPlots (please see Lord et al., JCB 2020) to better display the distribution of the entire dataset, and report statistics (such as means, error bars, and P values) that address the reproducibility of the findings.

Statistical methods should be explained in full in the materials and methods. For figures presenting pooled data the statistical measure should be defined in the figure legends. Please also be sure to indicate the statistical tests used in each of your experiments (both in the figure legend itself and in a separate methods section) as well as the parameters of the test (for example, if you ran a t-test, please indicate if it was one- or two-sided, etc.). Also, if you used parametric tests, please indicate if the data distribution was tested for normality (and if so, how). If not, you must state something to the effect that "Data distribution was assumed to be normal but this was not formally tested."

4) Title: The title should be less than 100 characters including spaces and accessible to a general readership. We feel that the mention of DT40 cells makes it sound like a narrow advance and may limit appeal. To convey the advance more clearly, we suggest the following title:
"Quantitative measurements of kinetochore protein copy numbers via FCS-calibrated molecular counting."

5) Materials and methods: Should be comprehensive and not simply reference a previous publication for details on how an experiment was performed. Please provide full descriptions (at least in brief) in the text for readers who may not have access to referenced manuscripts. The text should not refer to methods "...as previously described." Please also indicate the acquisition and quantification methods for immunoblotting/western blots.

6) For all cell lines, vectors, strains, constructs/cDNAs, etc. - all genetic material: please include database / vendor ID (e.g. Addgene, ATCC, etc.) or if unavailable, please briefly describe their basic genetic features, even if described in other published work or gifted to you by other investigators (and provide references where appropriate). Please be sure to provide the sequences for all of your oligos: primers, si/shRNA, RNAi, gRNAs, etc. in the materials and methods. You must also indicate in the methods the source, species, and catalog numbers/vendor identifiers (where appropriate) for all of your antibodies, including secondary. If antibodies are not commercial, please add a reference citation if possible.

7) Microscope image acquisition: The following information must be provided about the acquisition and processing of images:
a. Make and model of microscope
b. Type, magnification, and numerical aperture of the objective lenses
c. Temperature
d. Imaging medium
e. Fluorochromes
f. Camera make and model
g. Acquisition software
h. Any software used for image processing subsequent to data acquisition. Please include details and types of operations involved (e.g., type of deconvolution, 3D reconstitutions, surface or volume rendering, gamma adjustments, etc.).

8) References: There is no limit to the number of references cited in a manuscript. References should be cited parenthetically in the text by author and year of publication. Abbreviate the names of journals according to PubMed.

9) Supplemental materials: Reports are generally allowed 3 supplemental figures and 10 videos. You currently exceed this limit but, in this case, we will be able to give you the extra space. Please also note that tables, like figures, should be provided as individual, editable files. A summary of all supplemental material should appear at the end of the Materials and methods section. Please include one brief sentence per item.

10) Video legends: Should describe what is being shown, the cell type or tissue being viewed (including relevant cell treatments, concentration and duration, or transfection), the imaging method (e.g., time-lapse epifluorescence microscopy), what each color represents, how often frames were collected, the frames/second display rate, and the number of any figure that has related video stills or images.

11) eTOC summary: A ~40-50 word summary that describes the context and significance of the findings for a general readership should be included on the title page. The statement should be written in the present tense and refer to the work in the third person. It should begin with "First author name(s) et al..." to match our preferred style.

12) Conflict of interest statement: JCB requires inclusion of a statement in the acknowledgements regarding competing financial interests. If no competing financial interests exist, please include the following statement: "The authors declare no competing financial interests." If competing interests are declared, please follow your statement of these competing interests with the following statement: "The authors declare no further competing financial interests."

13) A separate author contribution section is required following the Acknowledgments in all research manuscripts. All authors should be mentioned and designated by their first and middle initials and full surnames. We encourage use of the CRediT nomenclature (https://casrai.org/credit/).

14) ORCID IDs: ORCID IDs are unique identifiers allowing researchers to create a record of their various scholarly contributions in a single place. Please note that ORCID IDs are required for all authors. At resubmission of your final files, please be sure to provide your ORCID ID and those of all co-authors.

15) JCB requires authors to submit Source Data used to generate figures containing gels and Western blots with all revised manuscripts. This Source Data consists of fully uncropped and unprocessed images for each gel/blot displayed in the main and supplemental figures. For assays performed using capillary electrophoresis and/or immunoassay-based detection, authors should instead provide the electropherogram graph(s) for each experiment, plotting fluorescence/chemiluminescence intensity vs. molecular weight/size. Since your paper includes cropped gel and/or blot images, please be sure to provide one Source Data file for each figure gels, blots, and/or capillary electrophoresis assays along with your revised manuscript files. File names for Source Data figures should be alphanumeric without any spaces or special characters (i.e., SourceDataF#, where F# refers to the associated main figure number or SourceDataFS# for those associated with Supplementary figures). For traditional gels and blots, the lanes of the gels/blots should be labeled as they are in the associated figure, the place where cropping was applied should be marked (with a box), and molecular weight/size standards should be labeled wherever possible. For capillary electrophoresis assays, each trace in the graph should be color-coded and labeled to indicate which protein, gene, or sample is being measured (please try to avoid red/green combinations to accommodate our color-blind readers).

Source Data files will be directly linked to specific figures in the published article. Source Data Figures should be provided as individual PDF files (one file per figure). Authors should endeavor to retain a minimum resolution of 300 dpi or pixels per inch. Please review our instructions for export from Photoshop, Illustrator, and PowerPoint here: https://rupress.org/jcb/pages/submission-guidelines#revised.

16) Journal of Cell Biology now requires a data availability statement for all research article submissions. These statements will be published in the article directly above the Acknowledgments. The statement should address all data underlying the research presented in the manuscript. Please visit the JCB instructions for authors for guidelines and examples of statements at (https://rupress.org/jcb/pages/editorial-policies#data-availability-statement).

B. FINAL FILES:

Please upload the following materials to our online submission system. These items are required prior to acceptance. If you have any questions, contact JCB's Managing Editor, Lindsey Hollander (lhollander@rockefeller.edu).

-- An editable version of the final text (.DOC or .DOCX) is needed for copyediting (no PDFs).

-- High-resolution figure and MP4 video files: See our detailed guidelines for preparing your production-ready images, https://jcb.rupress.org/fig-vid-guidelines.

-- Cover images: If you have any striking images related to this story, we would be happy to consider them for inclusion on the journal cover. Submitted images may also be chosen for highlighting on the journal table of contents or JCB homepage carousel. Images should be uploaded as TIFF or EPS files and must be at least 300 dpi resolution.

It is JCB policy that if requested, original data images must be made available to the editors. Failure to provide original images upon request will result in unavoidable delays in publication. Please ensure that you have access to all original data images prior to final submission.

The license to publish form must be signed before your manuscript can be sent to production. A link to the license to publish form will be sent to the corresponding author only. Please take a moment to check your funder requirements before choosing the appropriate license.

Additionally, JCB encourages authors to submit a short video summary of their work. These videos are intended to convey the main messages of the study to a non-specialist, scientific audience. Think of them as an extended version of your abstract, or a short poster presentation. We encourage first authors to present the results to increase their visibility. The videos will be shared on social media to promote your work. For more detailed guidelines and tips on preparing your video, please visit https://rupress.org/jcb/pages/submission-guidelines#videoSummaries.

Thank you for your attention to these final processing requirements. Please revise and format the manuscript and upload materials within 14 days. If you need an extension for whatever reason, please let us know and we can work with you to determine a suitable revision period.

Please contact the journal office with any questions at cellbio@rockefeller.edu.

Thank you for this interesting contribution, we look forward to publishing your paper in Journal of Cell Biology.

Sincerely,

Arshad Desai, PhD
Monitoring Editor
Journal of Cell Biology

Dan Simon, PhD
Scientific Editor
Journal of Cell Biology

Reviewer #1 (Comments to the Authors (Required)):

The authors have comprehensively addressed my comments. The manuscript should be published as a resource in JCB.

Reviewer #2 (Comments to the Authors (Required)):

The authors have addressed all my major concerns and the manuscript can be published in its current form.

Reviewer #3 (Comments to the Authors (Required)):

I thank the authors for their revisions. They answered my questions and I have no further comments.
