# Supplementary material for: Quantitative measurements of kinetochore protein copy numbers via FCS-calibrated molecular counting
Source: J Cell Biol. 2026 Nov 11;226(1):e202603185. doi: 10.1083/jcb.202603185 (PMC13753873; doi:10.1083/jcb.202603185)

Source Data FS4

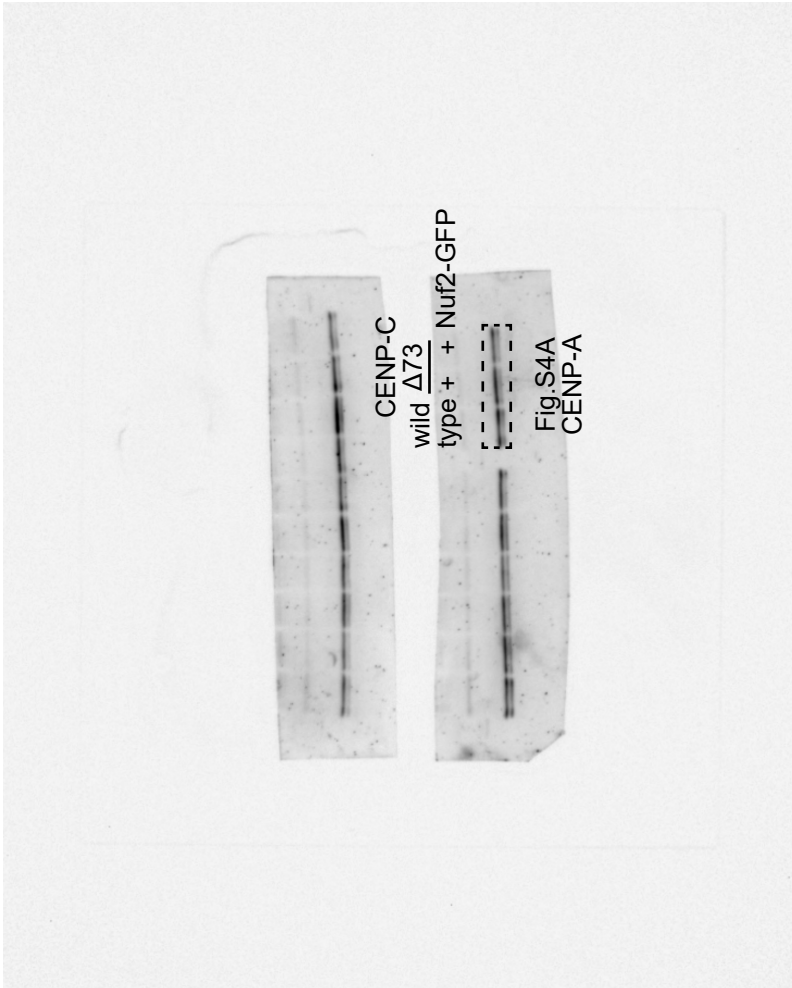

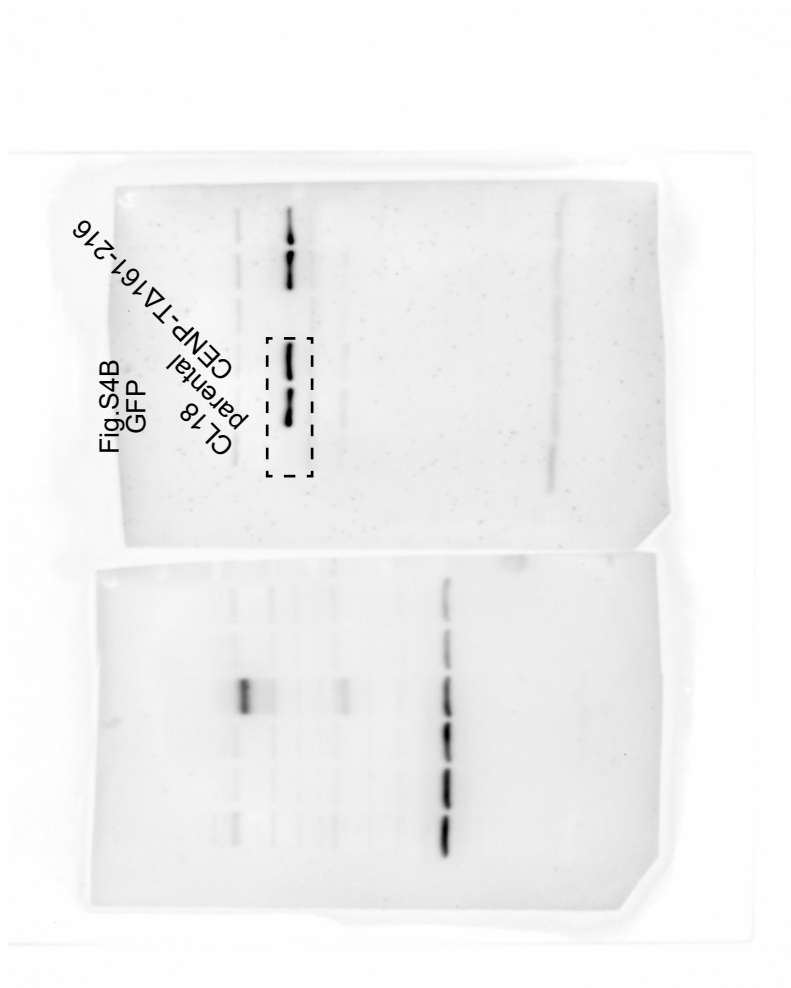

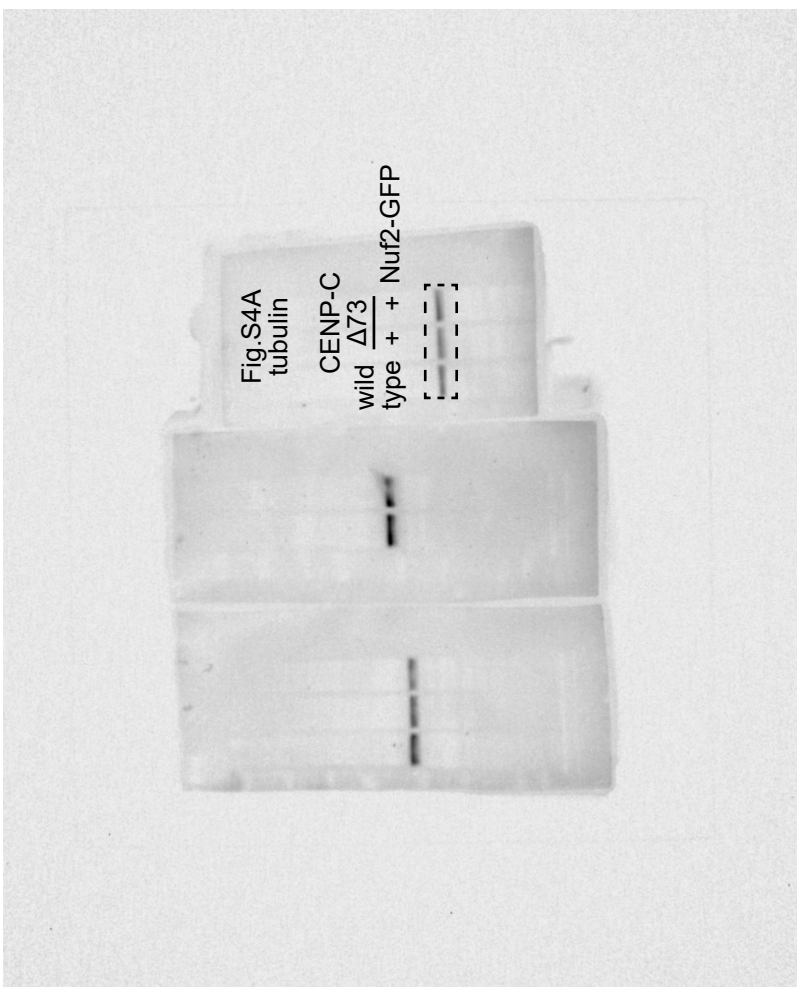

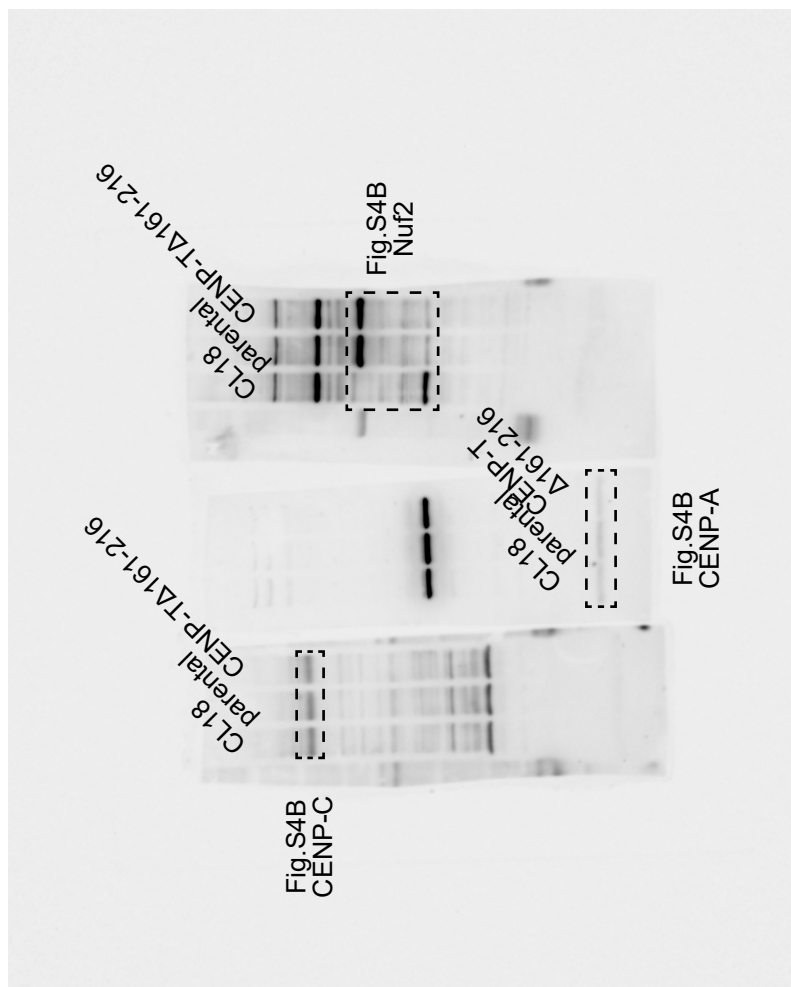

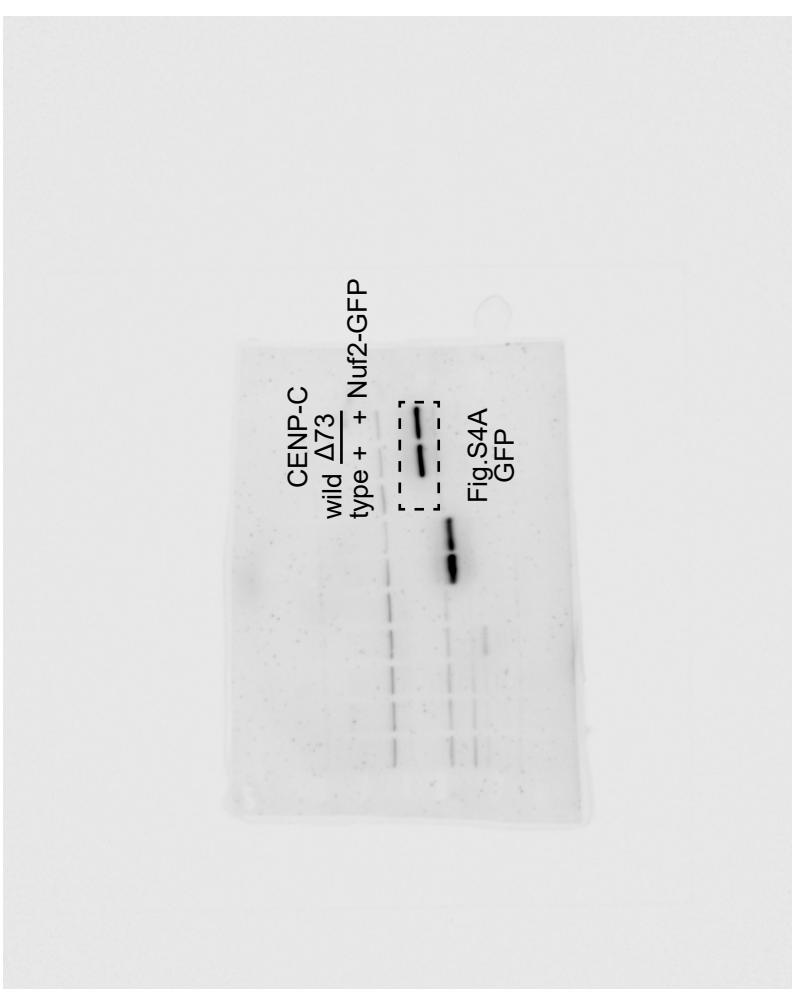

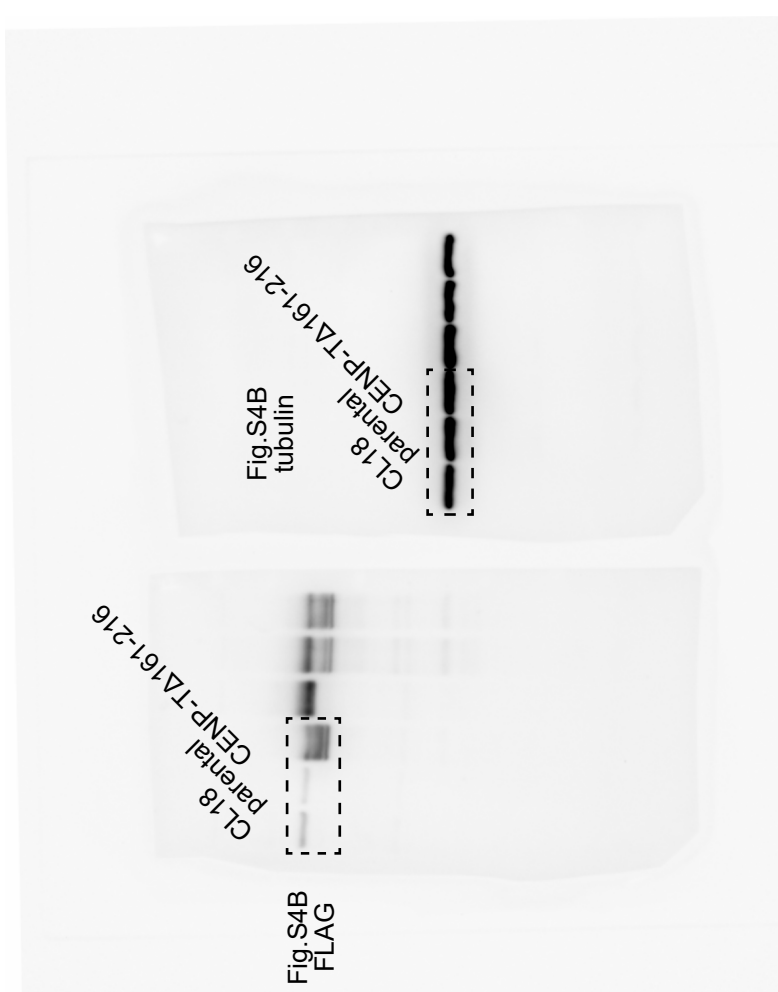

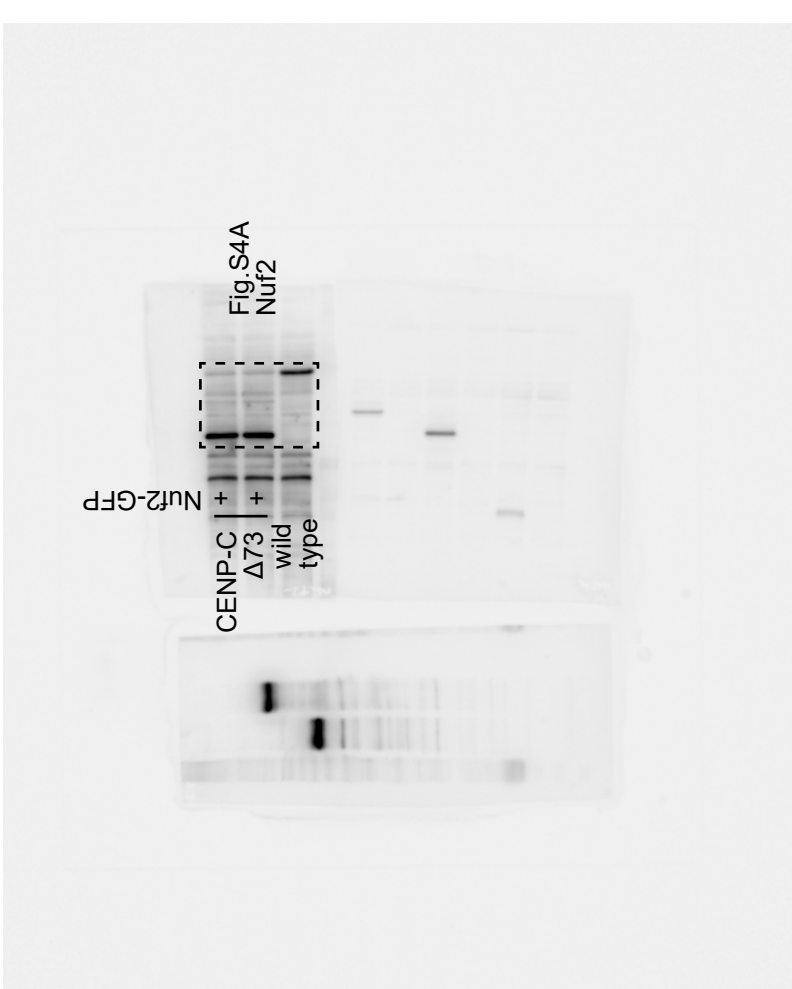

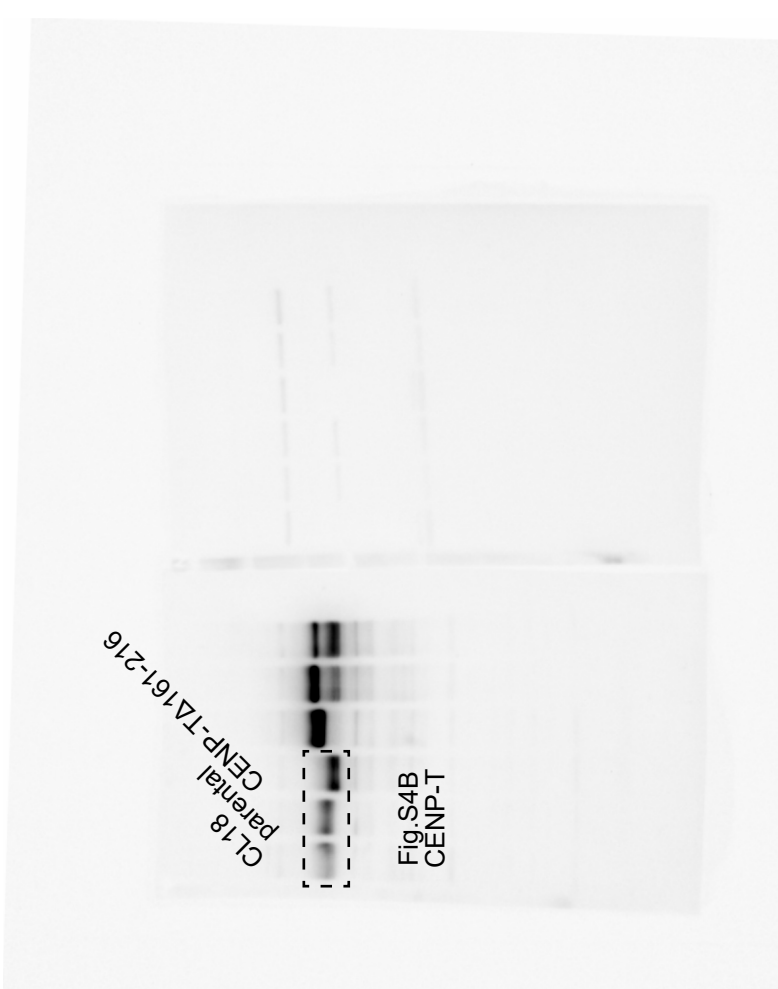

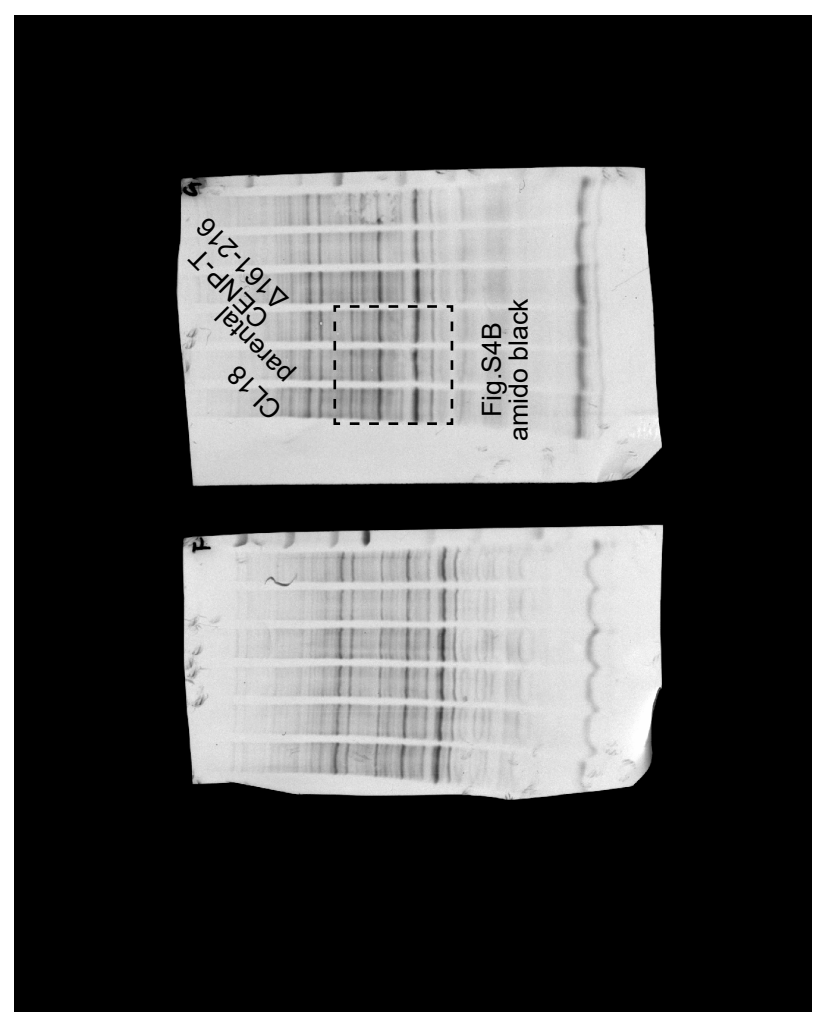

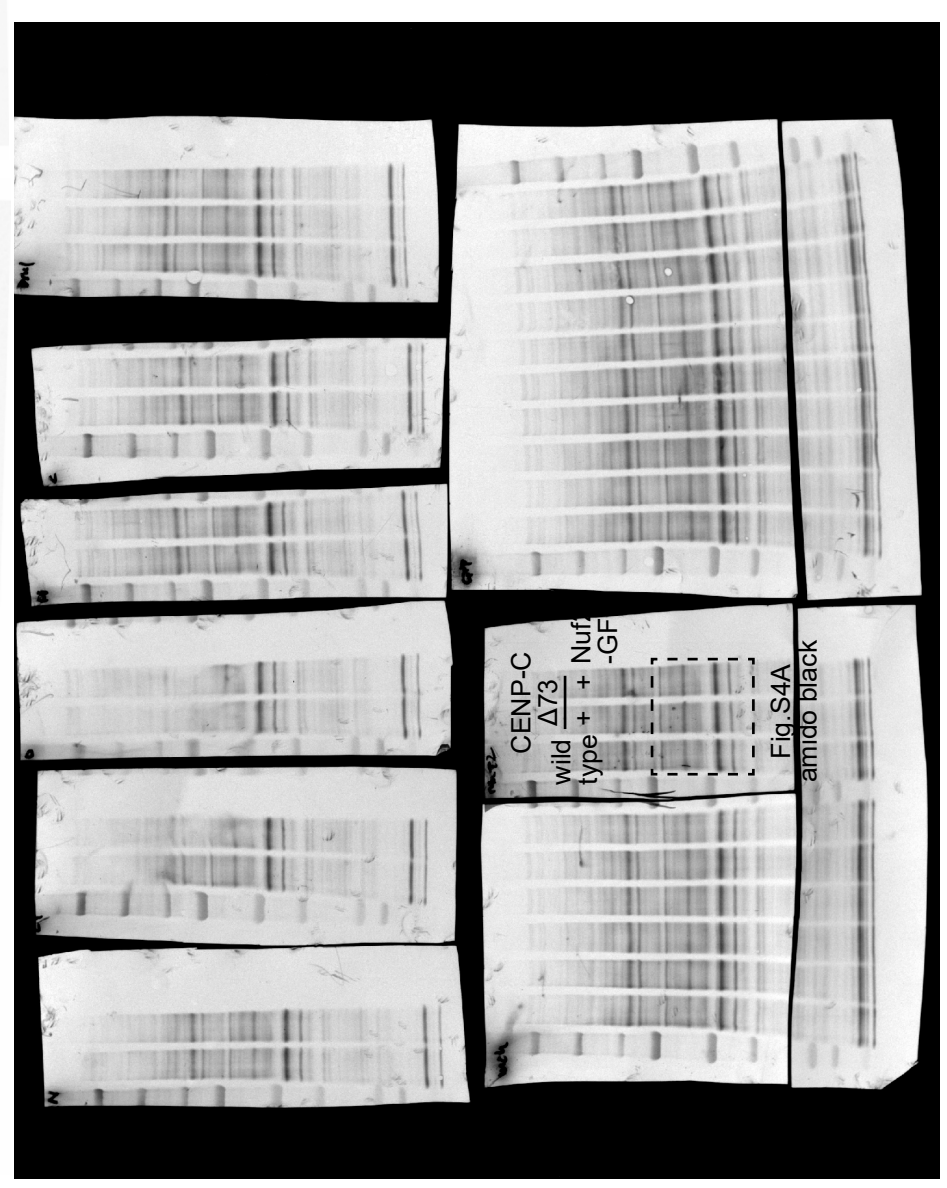

Supplement: Supplementary file 4 [file jcb_202603185_sourcedatafs4.pdf]
