# Supplementary material for: Quantitative measurements of kinetochore protein copy numbers via FCS-calibrated molecular counting
Source: J Cell Biol. 2026 Nov 11;226(1):e202603185. doi: 10.1083/jcb.202603185 (PMC13753873; doi:10.1083/jcb.202603185)
Supplement: Supplementary file 5 [file jcb_202603185_tables1.docx]

**Table S1.** DT40 strains used in this study

| Strain | Source |
| --- | --- |
| CL18 | Buerstedde *et al.*, EMBO J., 1990 (Fukagawa Lab. Stock) |
| CL18 / CENP-C-EGFP | Watanabe *et al.*, J. Cell Biol., 2019 |
| CL18 / mScarlet-HA-SNAP-CENP-T | Sakai *et al.*, Sci. Adv., 2026 |
| cKO-CENP-C/mScarlet-CENP-N | Hara *et al.*, Mol Cell, 2023 |
| CL18 / CENP-H-EGFP | Fukagawa *et al.*, EMBO J., 2001 |
| ΔCENP-O/ mScarlet-AID-CENP-O | This study |
| CL18 / EGFP-Dsn1 | Takenoshita *et al.*, iScience., 2024 |
| CL18 / EGFP-Dsn1 mCherry-CENP-T | This study |
| CENP-CΔ73 / GFP-Dsn1 | Takenoshita *et al.*, Nat. Commun., 2022 |
| CENP-CΔ73 / EGFP-Dsn1 mCherry-CENP-T | This study |
| CENP-TΔ161-216 / EGFP-Dsn1 | This study |
| CL18 / Nuf2-EGFP | This study |
| CENP-CΔ73 / Nuf2-EGFP | Takenoshita *et al.*, Nat. Commun., 2022 |
| CENP-TΔ161-216 / Nuf2-EGFP | This study |
