# Supplementary material for: Quantitative measurements of kinetochore protein copy numbers via FCS-calibrated molecular counting
Source: J Cell Biol. 2026 Nov 11;226(1):e202603185. doi: 10.1083/jcb.202603185 (PMC13753873; doi:10.1083/jcb.202603185)
Supplement: Supplementary file 6 [file jcb_202603185_tables2.docx]

**Table S2.** Plasmids used in this study

| Plasmids | Source | Oligos for construction (5’→3’) | |
| --- | --- | --- | --- |
| pYH77 / pBSSK_CT-N KI_mCherry-CENP-T FL_SV40-HisD | This study | F: ATGGTGAGCAAGGGCGAGGA  R: CTTGTACAGCTCGTCCATGCCG | |
| pYH78 / pBSSK_CT-N KI_mCherry-CENP-T FL_SV40-NeoR | This study | F: ATGGTGAGCAAGGGCGAGGA  R: CTTGTACAGCTCGTCCATGCCG | |
| pYT334 / pX330_ggCENP-T | Takenoshita *et al.*, iScience., 2024 | N/A |  |
| pBSKS_Nuf2 KI_GFP-Nuf2_PGK Puro^R^ | Takenoshita *et al.*, Nat. Commun., 2022 | N/A |  |
| pBSKS_Nuf2 KI_GFP-Nuf2_PGK EcoGPT | Takenoshita *et al.*, Nat. Commun., 2022 | N/A |  |
| pX330_ggNuf2 | Takenoshita *et al.*, Nat. Commun., 2022 | N/A |  |
| pYT331 / pBSSK_CT-N KI_FLAG-CENP-TΔ161-216_SV40- NeoR | Takenoshita *et al.*, iScience., 2024 | N/A |  |
| pYT448 / pBSSK_CT-N KI_FLAG-CENP-TΔ161-216_SV40-HisD | Takenoshita *et al.*, iScience., 2024 | N/A |  |
| pBSSK_ACTB KI_ OsTIR1-T2A-BSR_IRES_ mScarlet-AID-CENP-O | This study | F: CCTCCGCAGCCAGCCATGACATACTTTCCTGAAGAGGTCGT  R: TGTCCAAACTCATCAATGTATCTTAATGGATGATGATATT |  |
| pX335_ggACTB | Takenoshita *et al.*, Nat. Commun., 2022 | N/A |  |
| Nuf2-mCherry | This study | F: CGCGGGCCCGGGATCATGGAGGCGCTCACCTTTCCCCGCT  R: GGCGACCGGTGGATCAGAACTACTTCCAGGACTCTGAACT | |
| pYH1 / mScalet-EGFP | This study | F: GGTACCGCGGGCCCGGGATCCATGGTGAGCAAGGGCGAG  R: TAGATCCGGTGGATCCTTACTTGTACAGCTCGTCCATGCC | |
| EGFP-mCherry | This study | F: CGAGCTCAAGCTTCGAATTCTATGGTGAGCAAGGGCGAGGA  R: TAGATCCGGTGGATCCTTACTTGTACAGCTCGTCCATGC | |
|  |  |  | |
